# The critical role of Atpif1 in Her2-targeted CAR-T cell therapy for solid tumor via modulation of metabolism and mtDNA-STING signal pathway

**DOI:** 10.3389/fimmu.2026.1733753

**Published:** 2026-03-02

**Authors:** Genshen Zhong, Biao Liu, Xue Gong, Qi Wang, Shuyin Zheng, Xinyu Luo, Zhiguo Chen, Xingya Zhang, Biliang Hu, Minna Wu

**Affiliations:** 1 College of Biological and Chemical Engineering, Changsha University, Changsha, Hunan, China; 2 Changsha Technology Innovation Center for Immune Regulation and Molecular Targeted Drugs, Changsha University, Changsha, Hunan, China; 3 Henan Key Laboratory of Immunology and Targeted Therapy, School of Medical Technology, Henan Medical University, Xinxiang, Henan, China; 4 Hunan Siweikang Pharmaceutical Co., Ltd., Changsha, Hunan, China

**Keywords:** ATPIF1, CAR-T, hypoxia, mtDNA, STING

## Abstract

**Background:**

Chimeric Antigen Receptor-T (CAR-T) cell therapy has demonstrated remarkable success in hematological malignancies but remains limited in the treatment of solid tumors. This study investigates the role of ATP synthase inhibitory factor 1 (ATPIF1) in modulating the efficacy of Her2-targeted CAR-T cells against solid tumors through metabolic reprogramming and the mitochondrial DNA (mtDNA)-stimulator of interferon genes (STING) signaling pathway.

**Methods:**

Her2-targeted CAR-T cells with ATPIF1 overexpression (Her2-IF1 CAR-T) or knockdown (Her2-shIF1 CAR-T) were generated, and their antitumor activity was evaluated *in vitro* and *in vivo*. The underlying mechanisms were also elucidated.

**Results:**

*In vitro*, ATPIF1 overexpression enhanced CAR-T cell function, including increased tumor lysis, cytokine secretion (IL-2, IFN-γ), and oxidative phosphorylation (OCR). Conversely, ATPIF1 knockdown impaired these functions. Surprisingly, *in vivo* results revealed the opposite trend: Her2-shIF1 CAR-T cells exhibited superior tumor inhibition, while Her2-IF1 CAR-T cells showed reduced efficacy despite their prolonged persistence. Mechanistically, ATPIF1 knockdown increased mitochondrial membrane potential (MMP), promoted survival under hypoxic conditions (1% O_2_), and enhanced CAR-T infiltration into tumors. This was linked to mitochondrial permeability transition pore (mPTP) opening and mtDNA leakage, which activated the STING pathway, further amplifying T cell migration and antitumor responses. Inhibition of STING with H151 reversed these effects, confirming its critical role in modulating ATPIF1-mediated functions in Her2-targeting CAR-T cells.

**Conclusion:**

Our findings highlight the dual role of ATPIF1 in CAR-T cell therapy: while its overexpression boosts metabolic activity *in vitro*, its knockdown enhances adaptability to the hypoxic tumor microenvironment *in vivo*, indicating the paradox for modulating the antitumor activities of CAR-T cells via the metabolic remodeling for the treatment of solid tumor. These insights suggest that targeting ATPIF1 or the STING pathway could optimize CAR-T cell efficacy in solid tumors, bridging the gap between *in vitro* performance and *in vivo* outcomes.

## 1 Introduction

Over the past few decades, Chimeric Antigen Receptor-T (CAR-T) cells have demonstrated promising clinical efficacy in the treatment of hematological malignancies. Specifically, CAR-T cells targeting CD19 and BCMA antigens have been approved for clinical use in relapsed/refractory B-cell acute lymphoblastic leukemia (B-ALL), diffuse large B-cell lymphoma, and multiple myeloma, among other indications (1, 2). However, the efficacy of CAR-T cells in solid tumors has yet to live up to expectations (2, 3). This is primarily attributed to the hostile tumor microenvironment (TME), tumor antigen heterogeneity, insufficient trafficking and infiltration, as well as T-cell exhaustion and impaired persistence—all of which are detrimental factors that compromise the *in vivo* antitumor efficacy of CAR-T cells (2, 3). As the primary functional effectors, enhancing the antitumor activity of CAR-T cells is therefore crucial. Emerging research indicates that strategies such as modulating metabolism to induce a memory-like phenotype in CAR-T cells, alleviating T-cell exhaustion, and improving their persistence and tumor infiltration capacity have proven effective. Among these approaches, regulating mitochondrial metabolic plasticity and maintaining metabolic homeostasis are particularly critical (1, 3, 4).

Mitochondria (MT), the powerhouses of cellular energy metabolism, play a pivotal role in determining the immune differentiation phenotype of T cells (5). Their quality and metabolic functional status directly influence the *in vivo* persistence and antitumor activity of T cells. As dynamic double-membrane-bound organelles in eukaryotic cells, mitochondria are structurally divided into four functional compartments from the outer to the inner layer: the outer mitochondrial membrane (OMM), the intermembrane space, the inner mitochondrial membrane (IMM), and the mitochondrial matrix (6). Localized on the IMM, Complex V (also referred to as ATP synthase) is primarily responsible for ATP synthesis. Subsequent studies have demonstrated that ATP synthase not only mediates ATP synthesis but also catalyzes its hydrolysis, thereby exerting a bidirectional regulatory effect on intracellular ATP levels (7). The activity of ATP synthase is modulated by ATP synthase inhibitory factor 1 (ATPIF1, also designated as ATP5IF1)—the first identified nuclear-encoded, evolutionarily highly conserved mitochondrial protein that interacts with ATP synthase. By binding to the F1 domain of ATP synthase, ATPIF1 selectively inhibits either ATP synthesis or hydrolysis under distinct physiological and pathological conditions. Furthermore, ATPIF1 is closely implicated in mitochondrial cristae formation and heme biosynthesis, underscoring its vital role in the regulation of cellular energy metabolism (8, 9).

Our previous work has demonstrated that knockout of ATPIF1 impairs the antitumor activity of murine T cells, whereas overexpression of ATPIF1 can modulate the immune differentiation of CD19-targeted CAR-T cells (10). Specifically, ATPIF1 overexpression increases the proportion of CD8^+^ CAR-T cells, as well as the percentages of central memory T cells (T_CM_) and stem cell-like memory T cells (T_SCM_), which significantly prolongs the survival of NCG mice engrafted with CD19^+^ NALM-6 tumors. These findings indicate that ATPIF1 plays a pivotal role in regulating T cell metabolic reprogramming and immune differentiation, rendering it a potential molecular target for optimizing the antitumor efficacy of CAR-T cells. Notably, the observation that complete knockout of ATPIF1 compromises T cell function has recently been validated by the research team led by Dr. Cuezva JM, a Spanish scientist with long-standing expertise in ATPIF1 gene research (11).

Given that solid tumor treatment remains a critical unmet challenge in CAR-T cell research, we generated Her2-targeted CAR-T cells with modulated ATPIF1 expression (either overexpression or knockdown). We then systematically evaluated the antitumor efficacy of these ATPIF1-modulated Her2-targeted CAR-T cells. Intriguingly, our data revealed that ATPIF1 exerted opposing effects on regulating Her2-targeted CAR-T cell function *in vitro* versus *in vivo*. This finding highlights a key dilemma in CAR-T cell development: metabolic engineering strategies designed to enhance antitumor function may yield inconsistent outcomes under distinct oxygen microenvironments—such as the oxygen-rich bloodstream versus the hypoxic milieu of solid tumors.

## 2 Materials and methods

### 2.1 Cell lines

The human breast cancer cell line SKBR-3 (HTB-30, ATCC) was cultured in RPMI-1640 medium (Invitrogen, USA) supplemented with 10% fetal bovine serum (FBS) and 1% (v/v) penicillin-streptomycin (P/S; Sigma-Aldrich, USA) at 37 °C in a humidified atmosphere containing 5% CO_2_. The Her2^+^SKBR-3 cell line expressing luciferase (Her2^+^SKBR3-luc) was generated by stably transducing wild-type Her2^+^SKBR-3 cells with firefly luciferase.

### 2.2 Animals

The animal experimental protocols in this study were approved by the Institutional Animal Care and Use Committee (IACUC) of Changsha University and were conducted in strict compliance with its ethical guidelines and regulatory requirements. This study was performed under the Animal Ethics Approval No. CCSU-2023-014. For the euthanasia of the mice, mice were placed in an induction chamber for 2~3 minutes using a 30% volume per minute displacement rate of 100% CO_2_ to terminate the experiment. Then the samples were collected as needs.

### 2.3 Construction of HER2-targeted CAR recombinant plasmid and the CAR lentivirus

**Figure 1:**
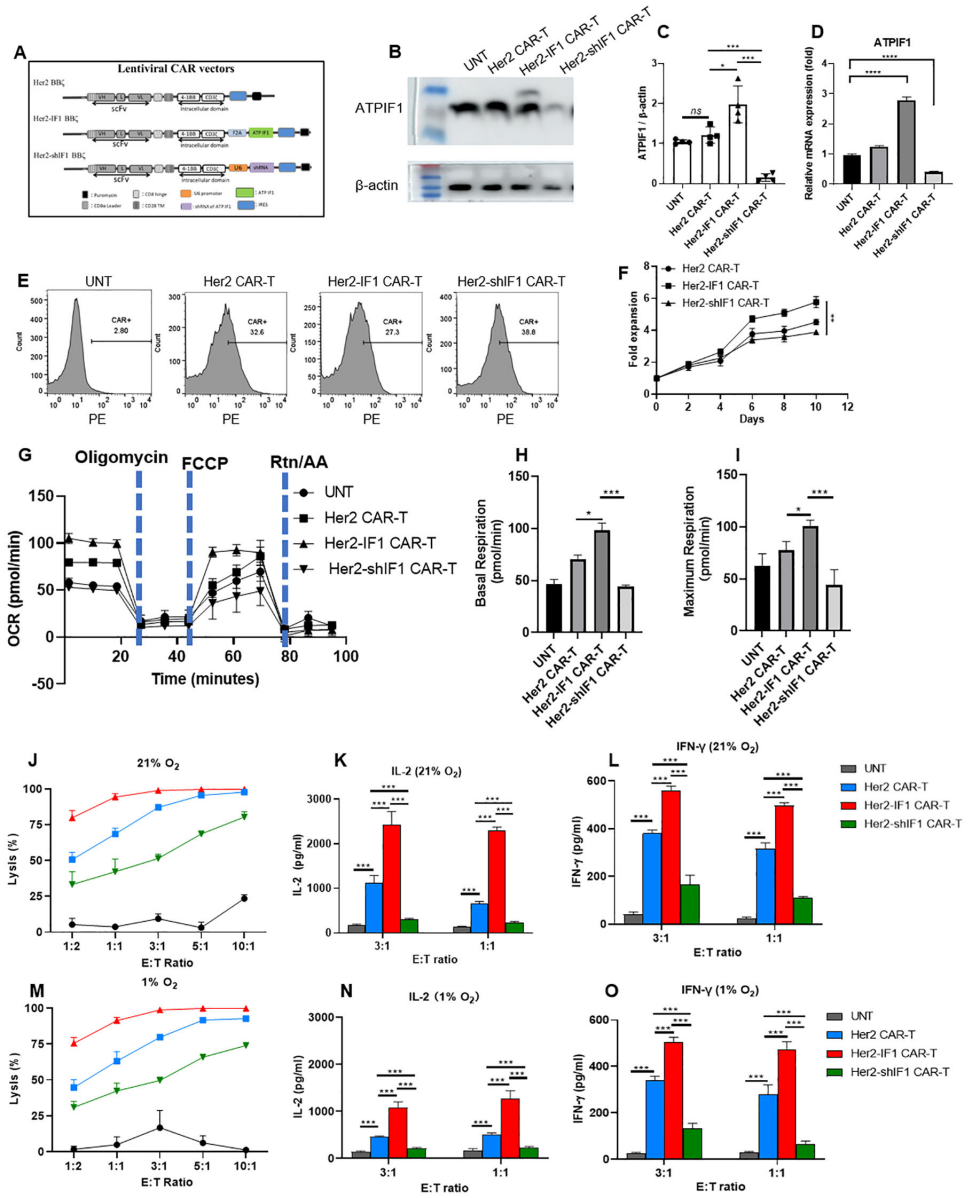
ATPIF1 knockdown impairs *in vitro* antitumor activity and rewires the metabolism of CAR-T cells. **(A)** Schematic diagrams of lentiviral vector constructs for Her2 CAR-T, Her2-IF1 CAR-T, and Her2-shIF1 CAR-T cells. a U6 promoter was inserted downstream of the Her2 CAR sequence to force the shRNA of ATPIF1. **(B, C)** ATPIF1 protein expression in CAR-T cells analyzed by Western blotting. **(D)** ATPIF1 mRNA levels in CAR-T cells determined by RT-qPCR. **(E)** Flow cytometric analysis of CAR-positive cells: CAR-T cells were first stained with biotin-labeled Her2 ectodomain protein, followed by incubation with PE-conjugated anti-biotin antibody for FACS detection of CAR^+^cells, the Y-axis was the count of cell numbers. **(F)***In vitro* proliferation of CAR-T cells. The Her2 CAR-T, Her2-IF1 CAR-T and Her2-shIF1 CAR-T cells were cultivated with IL-2 stimulation (50 IU/ml) for 12 days, the numbers of CAR^+^ cells were determined every two days. **(G-I)** Basal oxygen consumption rates (OCR) of resting Her2 CAR-T, Her2-IF1 CAR-T, and Her2-shIF1 CAR-T cells. Spare Respiratory Capacity (SRC) was calculated and plotted for both resting and activated cell states.**(J, M)** Cytolytic activity of untransfected T cells (UNT), Her2 CAR-T, Her2-IF1 CAR-T, and Her2-shIF1 CAR-T cells against SKBR3-Luc^+^cells at different effector-to-target (E:T) ratios following 24-hour co-culture under 21% O_2_ and 1% O_2_ conditions, respectively. **(K, N)** IL-2 concentration in co-culture supernatants from the experiment in **(J, M)**, respectively. **(L, O)** IFN-γ concentration in co-culture supernatants from the experiment in **(J, M)**, respectively. The ELISA method was used to determine the concentration of IL-2 and IFN-γ. *P < 0.05; **P < 0.01; ***P < 0.001; ****P < 0.0001..

DNA fragments encoding the single-chain variable fragment (scFv) anti-Her2 chimeric receptors (Her2 CAR or Her2-IF1 CAR) were synthesized by Genescript (Nanjing, China) and cloned into the lentiviral vector plenti-EF1A-puro to generate the Her2 CAR and Her2-IF1 CAR constructs. For the construction of Her2-shIF1 CAR, a U6 promoter was inserted downstream of the Her2 CAR sequence, as illustrated in **Figure 1A**. The ATPIF1-targeting shRNA sequences were as follows:

ATPIF1-shRNA-F: CCGGCCATGAAGAAGAAATCGTTCACTCGAGTGAACGATTTCTTCTTCATGGTTTTTG.

ATPIF1-shRNA-R: AATTCAAAAACCATGAAGAAGAAATCGTTCACTCGAGTGAACGATTTCTTCTTCATGG.

Lentivirus production for CAR constructs was performed as described in our previous publication.^10^.

### 2.4 CAR-T preparation

CD3+ T cells were isolated from the human Peripheral Blood Mononuclear Cell (PBMC) in preparation of the CAR-T cells. The experiment design was approved by the Medical Ethics Committee in Xinxiang Medical University, and the informed consent forms were signed by the donors before the blood collection.

PBMC was mixed with normal saline at a 1:1 ratio to dilute the blood. The diluted blood was then slowly layered onto lymphocyte separation medium (TBD, Tianjin, China) at a 1:3 ratio and centrifuged at 2000 rpm for 20 minutes. After centrifugation, a distinct white buffy coat layer was observed, which was carefully aspirated and transferred to a new centrifuge tube. An appropriate volume of PBS was added, followed by centrifugation at 2000 rpm for 8–10 minutes at room temperature. The supernatant was discarded, and this washing step was repeated twice. The resulting pellet contained PBMCs, which were resuspended in RPMI-1640 medium supplemented with 10% serum and thoroughly mixed by pipetting before cell counting.

T cells were isolated from the PBMCs using Human CD3 Positive Selection Kit (Cat. 8802-6830-74, Invitrogen). Purified T cells were stimulated with HuCD3/CD28/CD2 T cell Act (Cat. 10970, Stemcell) for 24 h. The T cells were then incubated in a complete RPMI 1640 medium containing hIL-2 (100 U/mL) and 10% FBS (Gibco) for expansion. On the following day, the T cells were infected with the lentivirus (Her2 CAR, Her2-IF1 CAR and Her2-shIF1 CAR) containing the target gene at a ratio of MOI = 5:1 of virus vs. cells. The transfected T lymphocytes were amplified and cultured in an incubator of 37 °C with 5% CO_2_ to obtain CAR-T cells. Five days after lentivirus infection, CAR+ cells were analyzed using the biotin-labeled Her2 protein (Cat: 10004-H02H, SinoBiological, China) following the PE-conjugated anti-biotin antibody (Cat: 12-9895-82, eBioscience).

For the cultivation of CAR-T under different O_2_ concentration, the CAR-T cells were placed in the conventional two gas incubator (21% O_2_), or placed in the three gas incubator (Thermofisher Heracell™ VIOS 160i), and the concentration of nitrogen is automatic adjusted to maintain the oxygen level at 1% (1% O_2_).

### 2.5 Real-time RT-PCR assay

Total RNAs were extracted from the CAR-T cells using TRIzol reagent (Takara Bio) and converted into cDNAs. Real-time PCR analysis was performed using SYBR Green Master Mix (Takara Bio). The ^ΔΔ^Ct method was used to calculate the gene expression levels after normalization to the GUSB. The primer information is listed in **Supplementary Table 1**.

### 2.6 Generation and confirmation of ρ^0^ CAR-T cells

The generation of ρ^0^cells has been described previously (12). To deplete mitochondrial DNA (mtDNA), CAR-T cells were cultured in RPMI-1640 medium supplemented with 10% fetal bovine serum (FBS), human IL-2 (100 U/mL), and ethidium bromide (EB, 25 μg/mL) for over 1 week. Subsequently, both EB-untreated control CAR-T cells and EB-treated CAR-T cells were harvested. Mitochondrial D-loop DNA was extracted and subjected to real-time RT-PCR analysis.

### 2.7 Western blot

Different types of CAR-T cells were lysed in RIPA buffer containing phosphatase inhibitors. Protein concentrations of samples were determined using a BCA assay and normalized. Samples were then denatured at 100 °C for 10 minutes.

Proteins were separated by electrophoresis on 10% or 15% SDS-polyacrylamide gels. After sample loading, electrophoresis was performed at 90 V for 20 minutes initially, then adjusted to 120 V until completion. Subsequently, proteins were transferred to a PVDF membrane at 260 mA for 90 minutes. The membrane was blocked with 5% skim milk for 1 hour at room temperature, followed by four washes with TBST (5 minutes per wash). Primary antibodies were incubated overnight at 4 °C, after which the membrane was washed four times with TBST (5 minutes each). Secondary antibodies were applied for 1 hour at room temperature, and the membrane was washed again four times with TBST (5 minutes per wash). Finally, protein bands were visualized using a chemiluminescence detection kit.

### 2.8 Flow cytometry analysis of CAR-T cell apoptosis

An appropriate number of CAR-T cells, which had been stimulated with SKBR3-Luc tumor cell-conditioned medium for 48 hours, were transferred to a 1.5 mL EP tube and centrifuged at 1300 rpm for 5 minutes. The cells were washed with PBS and then stained using an apoptosis detection kit (Cat# C1065L, Beyotime, China). After staining, the cells were incubated at 37 °C in the dark for 30 minutes. Following an additional PBS wash, each sample was resuspended in 300 µL of assay buffer and analyzed by flow cytometry within 1 hour. Data were processed using FlowJo software.

### 2.9 Quantification of mtDNA

The amount of mtDNA was quantified as previously described (13). Briefly, CAR-T cells (1 × 10^6^) were resuspended in 170 μl of digitonin buffer containing 150 mM NaCl, 50 mM HEPES, pH 7.4 and 25 μg/ml digitonin (HY-N4000, MedChemExpress). The homogenates were incubated on a rotator for 10 min at room temperature, followed by centrifugation at 15, 000g for 25 min at 4 °C. A 1:20 dilution of the supernatant (cmtDNA) was used for qPCR. The pellet was resuspended in 340 μl of lysis buffer containing 5 mM EDTA and proteinase K (HY-108717, MedChemExpress) and incubated at 55 °C overnight. The digested pellet was diluted with water (1:20 to 1:100) and heated at 95 °C for 20 min to inactivate proteinase K and the sample was used for qPCR with mtDNA-specific primers listed in **Supplementary Table 1**. The cmtDNA in the supernatant was normalized to the β-actin in the pellet for each sample.

### 2.10 Animal experiments

For the persistence assay, tumor-free NCG mice were used. Her2 CAR-T and Her2-IF1 CAR-T cells were administered via tail vein injection, with simultaneous intraperitoneal injection of IL-2 (100, 000 IU per mouse, twice weekly). Two weeks later, mice were euthanized, and peripheral blood mononuclear cells (PBMCs) and splenocytes were collected. CAR-T cells were detected using flow cytometry.

For evaluation of antitumor activity, three independent antitumor experiments were performed. SKBR3 cells were resuspended in sterile saline to a density of 5×10^6^cells/mL and kept on ice for subsequent use. The cell suspension was gently mixed, and a disposable syringe was used to aspirate the suspension. Each NCG mouse (Cat# T001475, GemPharmatech, China) received a subcutaneous injection of 200 µL cell suspension in the axillary region. Approximately one month later, when axillary tumor volumes reached ~100 mm³, mice were randomly assigned to groups.

1. For assessing the antitumor activity of Her2 CAR-T and Her2-IF1 CAR-T cells, seventeen SKBR3-Luc tumor-bearing NCG mice were used. Untransfected T cells (UNT), Her2 CAR-T, and Her2-IF1 CAR-T cells were administered via tail vein injection at a dose of 1×10^6^CAR^+^cells per mouse. The total number of cells in Her2 CAR-T and Her2-IF1 CAR-T groups was adjusted to match that of the UNT group using additional UNT cells. Three weeks later, mice were euthanized, and tumors were excised and weighed.
2. For comparing the antitumor activity of Her2 CAR-T, Her2-IF1 CAR-T, and Her2-shIF1 CAR-T cells, twenty-two SKBR3-Luc tumor-bearing NCG mice were used. The procedure was identical to that described in the above (1).
3. For evaluating the antitumor activity of Her2 CAR-T, Her2-IF1 CAR-T, and Her2-shIF1 CAR-T cells with or without the STING inhibitor H151, forty SKBR3-Luc tumor-bearing NCG mice were used. H151 was administered at a dosage of 7 mg/kg, as previously described (14). The remaining procedure was identical to that in (1).

Throughout the experiments, mouse body weights were monitored, and tumor growth was recorded. Approximately 4 weeks later, NCG mice were euthanized, and axillary tumors were excised. Tumor size and weight in each group were measured and recorded.

### 2.11 ELISA

The supernatant of CAR-T cells co-cultured with SKBR3-Luc cells was collected. Concentrations of IL-2 (Cat# 88-7025-88, ThermoFisher) and IFN-γ (Cat# 88-7316-86, ThermoFisher) in the supernatant were determined using ELISA kits following the manufacturer’s instructions.

### 2.12 Mitochondria function

CAR-T cells were loaded at 1 x 10^6^/well into XF24 plate, which was pre-coated with Cell-Tak for 20 min to increase the adhesion of CAR-T cells. The determination of OCR (Oxygen Consumption Rate) of CAR-T cells was performed as described in our previous study (10).

### 2.13 Mitochondrial membrane potential and mitochondrial permeability transition pore determination

The CAR-T cells cultured under 21% O_2_ and 1% O_2_ concentration were collected, and were stained with biotin-labeled Her2 protein (Cat: 10004-H02H, SinoBiological, China) following the PE conjugated anti-biotin antibody (Cat: 12-9895-82, eBioscience), and with the addition of TMRE in the MMP assay kit (Cat# C2001S, Beyotime Biotechnology Limited Company, Shanghai, China) or the mPTP assay kit (Cat# C2009S, Beyotime Biotechnology Limited Company, Shanghai, China), the TMRM and mPTP was determined with flow cytometry (BD, FACSCalibur) and the results in CAR^+^ T cells were analyzed with the Flowjo software.

### 2.14 MitoTracker assay

The CAR-T cells cultured under 21% O_2_ and 1% O_2_ concentration were collected, and were stained with biotin-labeled Her2 protein (Cat: 10004-H02H, SinoBiological, China) following the Alexa Fluor™ anti-biotin antibody (Cat: 53-9895-82, eBioscience), after that the addition of Mito-Tracker Red CMXros (Beyotime, China, Cat#C1049B-250 μg), the mitochondrial mass was determined with flow cytometry (BD, FACSCalibur).

### 2.15 Multiplex immunohistochemistry

Organizational Chip of Her2^+^ breast cancer was purchased from the Shanghai Outdo Biotech Co., LTD (Outdo Biotech) was used for mIHC analysis, the Formalin‐Fixed Paraffin‐Embedded sections labelled with DAPI (blue), ATPIF1 (yellow), CD3 (green), HIF-1α (Orange) were scanned using the Vectra imaging system.

### 2.16 Transwell assay

CAR-T cells were stained with the Cell Tracker Red CMTPX live-cell tracking Dye (Cat# MX4109, Maokangbio, Shanghai, China). Different types of CAR-T cells were resuspended in RPMI-1640 complete medium and seeded into Transwell chambers at 200 µL per well. SKBR3-Luc conditioned medium (600 µL per well) was added to the lower chambers. After 4 hours of incubation, migrated CAR-T cells in the lower chambers were visualized using a fluorescence microscope. Five random fields of view were selected per well, imaged, and recorded for quantitative analysis.

### 2.17 Statistical analysis

Data are presented as mean ± standard deviation (SD). All *in vitro* experiments were repeated at least three times, and data were analyzed using an unpaired two-tailed t-test. For *in vivo* experiments, data were analyzed by *post hoc* tests following one-way analysis of variance (ANOVA) using the SPSS 20.0 software package. *P* value < 0.05 was considered statistically significant.

## 3 Results

### 3.1 ATPIF1 overexpression enhanced the antitumor function of Her2 CAR-T *in vitro*

**Figure 2:**
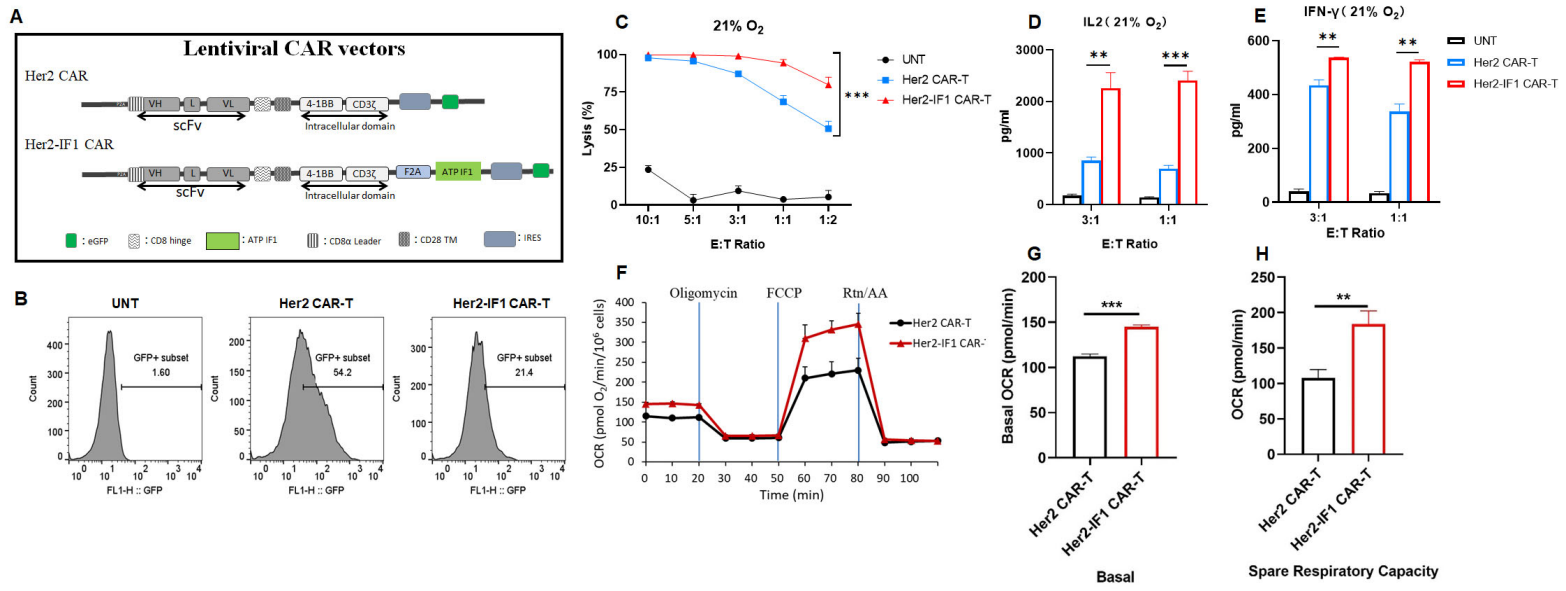
ATPIF1 overexpression enhances the *in vitro* antitumor activity and oxygen consumption rate (OCR) of Her2-targeted CAR-T cells. **(A)** Schematic diagrams of lentiviral vector constructs for Her2 CAR-T and Her2-IF1 CAR-T cells. The ATPIF1 gene is integrated in tandem after the Her2 CAR vector. **(B)** Flow cytometric identification of GFP-positive cells. **(C)** Cytolytic activity of untransfected T cells (UNT), Her2 CAR-T, and Her2-IF1 CAR-T cells against SKBR3-Luc^+^cells at different effector-to-target (E:T) ratios following 24-hour co-culture. Lytic function was evaluated via bioluminescent killing assay in 96-well microplates. Data are presented as mean ± standard deviation (SD) of triplicate wells from three independent experiments. **(D, E)** Cytokines determination. Levels of IL-2 **(D)** and IFN-γ **(E)** in co-culture supernatants, measured by enzyme-linked immunosorbent assay (ELISA). **(F–H)** OXPHOS determination with the Seahorse equipment. Basal OCR of resting Her2 CAR-T and Her2-IF1 CAR-T cells. Spare Respiratory Capacity (SRC) was calculated and plotted for both resting and activated cell states. **P<0.01; ***P<0.001.

Given that ATPIF1 overexpression has been shown to enhance the antitumor activity of αCD19 CAR-T cells both *in vitro* and *in vivo*, we engineered Her2-targeting CAR-T cells to overexpress ATPIF1 with the aim of improving their antitumor efficacy against solid tumors. As illustrated in **Figure 2A**, ATPIF1-overexpressing Her2-targeted CAR-T cells (Her2-IF1 CAR-T) were successfully generated, and this was validated by flow cytometric analysis of GFP expression (**Figure 2B**) and Western blotting (WB; **Supplementary Figure 1**). For *in vitro* experiments, Her2 CAR-T cells were co-cultured with luciferase-expressing breast cancer SKBR3 cells (SKBR3-Luc^+^). As depicted in **Figure 2C**, Her2-IF1 CAR-T cells exhibited more potent tumor lysis activity compared to parental Her2 CAR-T cells. Additionally, the levels of IFN-γ and IL-2 in the co-culture supernatants were significantly higher in the Her2-IF1 CAR-T group than in the Her2 CAR-T group (**Figures 2D, E**). These results indicate that ATPIF1 overexpression enhances the *in vitro* antitumor efficacy of Her2 CAR-T cells. To explore the underlying mechanism, a Seahorse extracellular flux analyzer was used to assess oxidative phosphorylation (OXPHOS) in Her2 and Her2-IF1 CAR-T cells. As shown in **Figures 2F–H**, the oxygen consumption rate (OCR) was markedly elevated in Her2-IF1 CAR-T cells, suggesting that ATPIF1 overexpression improves the mitochondrial respiratory chain function of CAR-T cells.

### 3.2 ATPIF1 overexpression prolonged the persistence of Her2-targeted CAR-T cells but impair the antitumor efficacy *in vivo*

**Figure 3:**
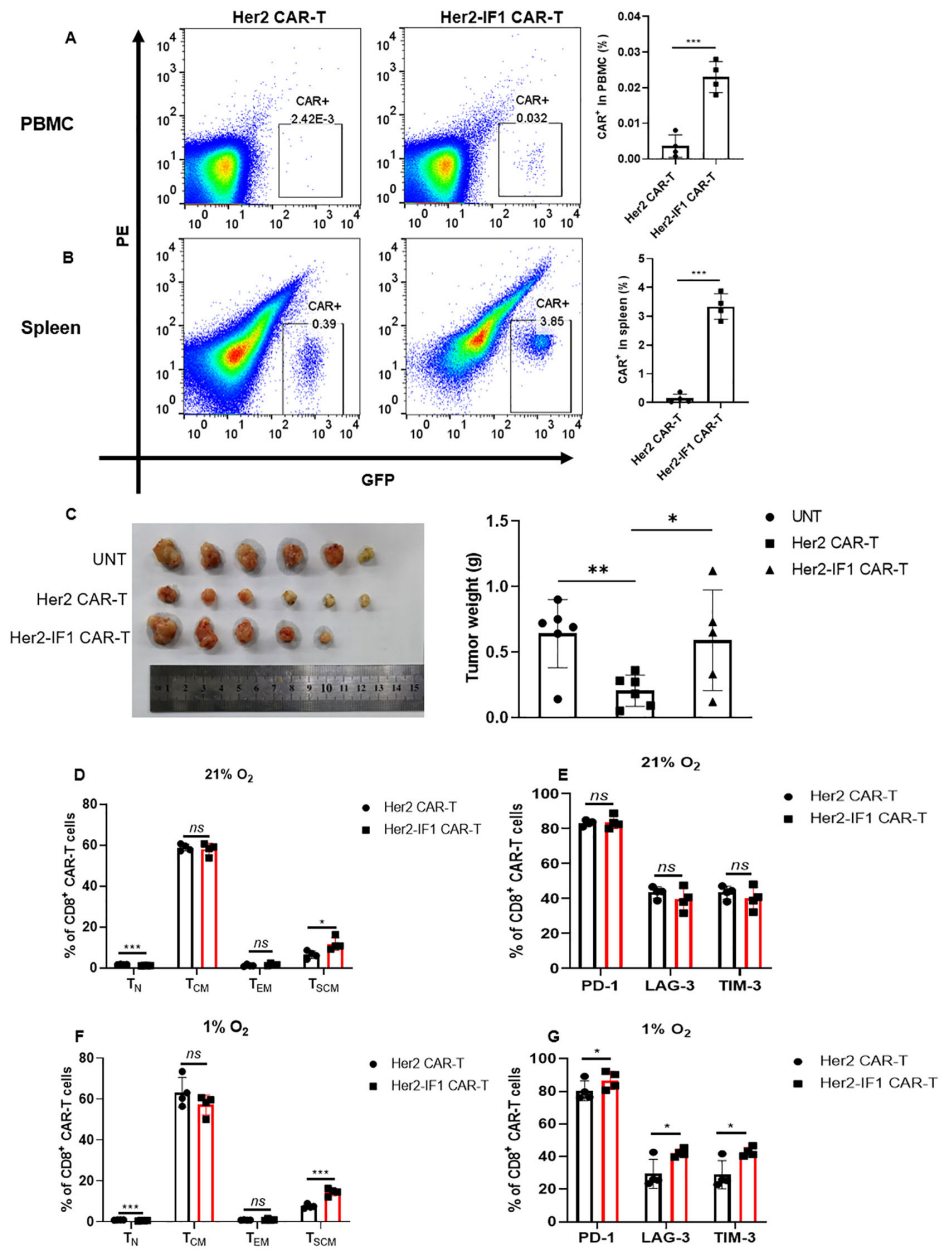
ATPIF1 overexpression enhances CAR-T cell persistence but impairs *in vivo* antitumor activity. **(A, B)** Flow cytometric quantification of GFP^+^Her2 CAR-T and Her2-IF1 CAR-T cells in mouse peripheral blood mononuclear cells (PBMCs) and spleens. NCG mice (n=4 in each group) were intravenously injected with Her2 CAR-T, Her2-IF1 CAR-T cells (1x10^6^ CAR-T cells per mouse). Three weeks later, the mice were euthanized, the peripheral blood and spleen were collected, respectively. The flow cytometry was used to detect the percentage of GFP^+^ CAR-T cells. **(C)** Tumor growth inhibition by Her2 CAR-T and Her2-IF1 CAR-T cells in SKBR-3 xenografted NCG mice. NCG mice were xenografted with SKBR3 cells (n=5 or 6 in each group). When tumors reached ~100 mm³, mice were intravenously injected with untransfected T cells (UNT), Her2 CAR-T, Her2-IF1 CAR-T cells (1x10^6^ CAR-T cells per mouse). Three weeks later, the mice were euthanized, tumors were excised and weighed. *P < 0.05; **P < 0.01. **(D, F)** The memory phenotype of Her2 CAR-T and Her2-IF1 CAR-T cells under 21% O_2_ and 1% O_2_ after stimulated with the conditional medium of SKBR-3 cells. **(E, G)** The exhaustion markers of PD-1, LAG-3 and TIM-3 determination in Her2 CAR-T and Her2-IF1 CAR-T cells under 21% O_2_ and 1% O_2_ after stimulated with the conditional medium of SKBR-3 cells. *P<0.05; **P<0.01; ***P<0.001.

The *in vivo* persistence of CAR-T cells is critical for their antitumor efficacy. Therefore, we assessed the impact of ATPIF1 overexpression on CAR-T cell persistence using flow cytometry. Nontumor-bearing mice were intravenously injected with equal numbers of CAR^+^Her2 CAR-T and Her2-IF1 CAR-T cells, and the number of CAR-T cells in peripheral blood mononuclear cells (PBMCs) and spleens was quantified two weeks post-injection. As shown in **Figures 3A, B**.

ATPIF1 overexpression significantly enhanced the *in vivo* persistence of CAR-T cells, the percentage of GFP^+^Her2-IF1 CAR-T cells was approximately 10-fold higher in both PBMCs and spleens compared to parental Her2 CAR-T cells. Surprisingly, however, the antitumor efficacy in mice xenografted with Her2^+^SKBR3 tumor cells was reversed (**Figure 3C**). Specifically, the tumor weight in the Her2-IF1 CAR-T treatment group was significantly greater than that in the Her2 CAR-T group, indicating that ATPIF1 overexpression impairs the *in vivo* antitumor efficacy of Her2 CAR-T cells. To elucidate the underlying mechanism, the memory phenotypes and exhaustion markers of CD8^+^ CAR-T cells were determined *in vitro* under 21% O_2_ and 1% O_2_ conditions, respectively. As shown in **Figures 3D, F**, there were no significant difference of Her2-IF1 CAR-T cells under different oxygen conditions, the ratio of different memory phenotype in CD8^+^ CAR-T was similar. However, the exhaustion markers of PD-1, LAG-3 and TIM-3 in Her2-IF1 CAR-T cells were significantly increased as compared to that of Her2 CAR-T cells under 1% O_2_ condition *in vitro*, indicating that ATPIF1 overexpression might increase the exhaustion of CD8^+^ CAR-T cells under hypoxic condition (**Figures 3E, G**).

### 3.3 Knockdown ATPIF1 impairs the antitumor activity of CAR-T cells *in vitro*

Given that ATPIF1 overexpression impaired the *in vivo* tumor-inhibitory efficacy of Her2 CAR-T cells, we further investigated the impact of ATPIF1 knockdown on Her2 CAR-T cell function. As illustrated in **Figures 1A–E**, both ATPIF1-overexpressing Her2-IF1 CAR-T cells and ATPIF1-knockdown Her2-shIF1 CAR-T cells were successfully constructed. Notably, Her2-shIF1 CAR-T cells exhibited the slowest proliferation rate among the three groups (**Figure 1F**). Consistent with our previous findings, Seahorse analysis revealed that the OCR was markedly elevated in Her2-IF1 CAR-T cells, whereas ATPIF1 knockdown reduced both the basal respiration and maximum respiratory capacity of CAR-T cells (**Figures 1G–I**). Meanwhile, Her2-IF1 CAR-T cells maintained enhanced tumor lysis activity compared to parental Her2 CAR-T cells, whereas ATPIF1 knockdown impaired the *in vitro* cytolytic capacity of CAR-T cells (**Figure 1J**). Also, Her2-IF1 CAR-T cells secreted significantly higher levels of IL-2 and IFN-γ than both Her2 CAR-T and Her2-shIF1 CAR-T cells (**Figure 1K, L**). In contrast, Her2-shIF1 CAR-T cells showed the lowest levels of IL-2 and IFN-γ secretion, as well as the weakest tumor lysis activity, compared to the other two groups (**Figures 1J–L**). Additionally, Her2-IF1 CAR-T cells still had the enhanced cytolytic lysis function and increased IL-2, IFN-γ secretion even under hypoxic condition (1% O_2_) (**Figures 1M–O**). These results indicate that ATPIF1 knockdown diminishes the *in vitro* antitumor function of Her2 CAR-T cells.

### 3.4 Her2-shIF1 CAR-T showed better tumor inhibitory efficacy *in vivo*

**Figure 4:**
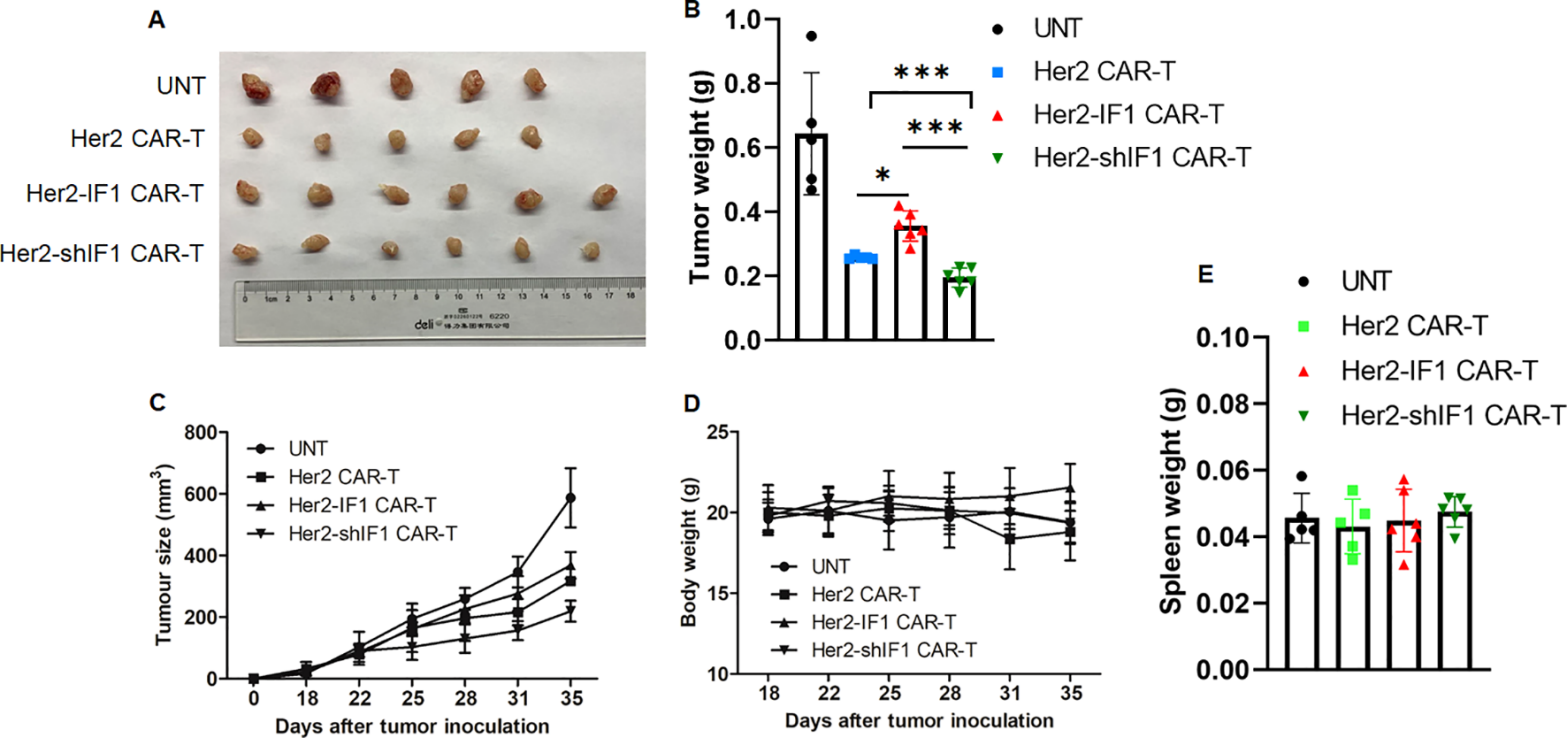
Her2-shIF1 CAR-T cells exhibit enhanced *in vivo* antitumor activity. **(A, B)***In vivo* tumor growth inhibition by UNT, Her2 CAR-T, Her2-IF1 CAR-T, and Her2-shIF1 CAR-T cells. NCG mice (n=5 or 6 in each group) were xenografted with SKBR3 cells. When tumors reached ~100 mm³, mice were intravenously injected with UNT, Her2 CAR-T, Her2-IF1 CAR-T cells (1x10^6^ CAR-T cells per mouse). Two weeks later, the mice were euthanized, tumors were excised and weighed. **(C)** Tumor growth curves of xenografted tumors in each treatment group, the tumor size is calculated with the formula 0.5*a*b^2^, a is the length of tumor and b is the width of tumor. **(D)** Body weight changes of mice during the experimental period. **(E)** Spleen weights of mice at the end of the experiment. *P < 0.05; ***P < 0.001.

To evaluate the *in vivo* antitumor activity of Her2 CAR-T, Her2-IF1 CAR-T, and Her2-shIF1 CAR-T cells, we conducted antitumor experiments using tumor-xenografted mice. As depicted in **Figures 4A–C**, tumor growth was significantly retarded following CAR-T cell administration. Surprisingly, Her2-shIF1 CAR-T cells exhibited the most potent inhibitory efficacy compared to that of untransfected T cells (UNT), Her2 CAR-T cells, and Her2-IF1 CAR-T cells. Notably, there were no significant differences in body weight or spleen weight among the various groups (**Figures 4D, E**). Consistent with the tumor growth curves, the tumor weight in the Her2-shIF1 CAR-T treatment group was the lowest (**Figure 4B**), indicating that ATPIF1 knockdown enhances the *in vivo* antitumor activity of Her2 CAR-T cells—despite its impairment of antitumor function *in vitro*. In contrast, ATPIF1 overexpression was found to diminish *in vivo* antitumor efficacy, which is consistent with the results presented in **Figure 3C**.

### 3.5 Her2-shIF1 CAR-T showed higher survivability under low oxygen condition *in vitro*

**Figure 5:**
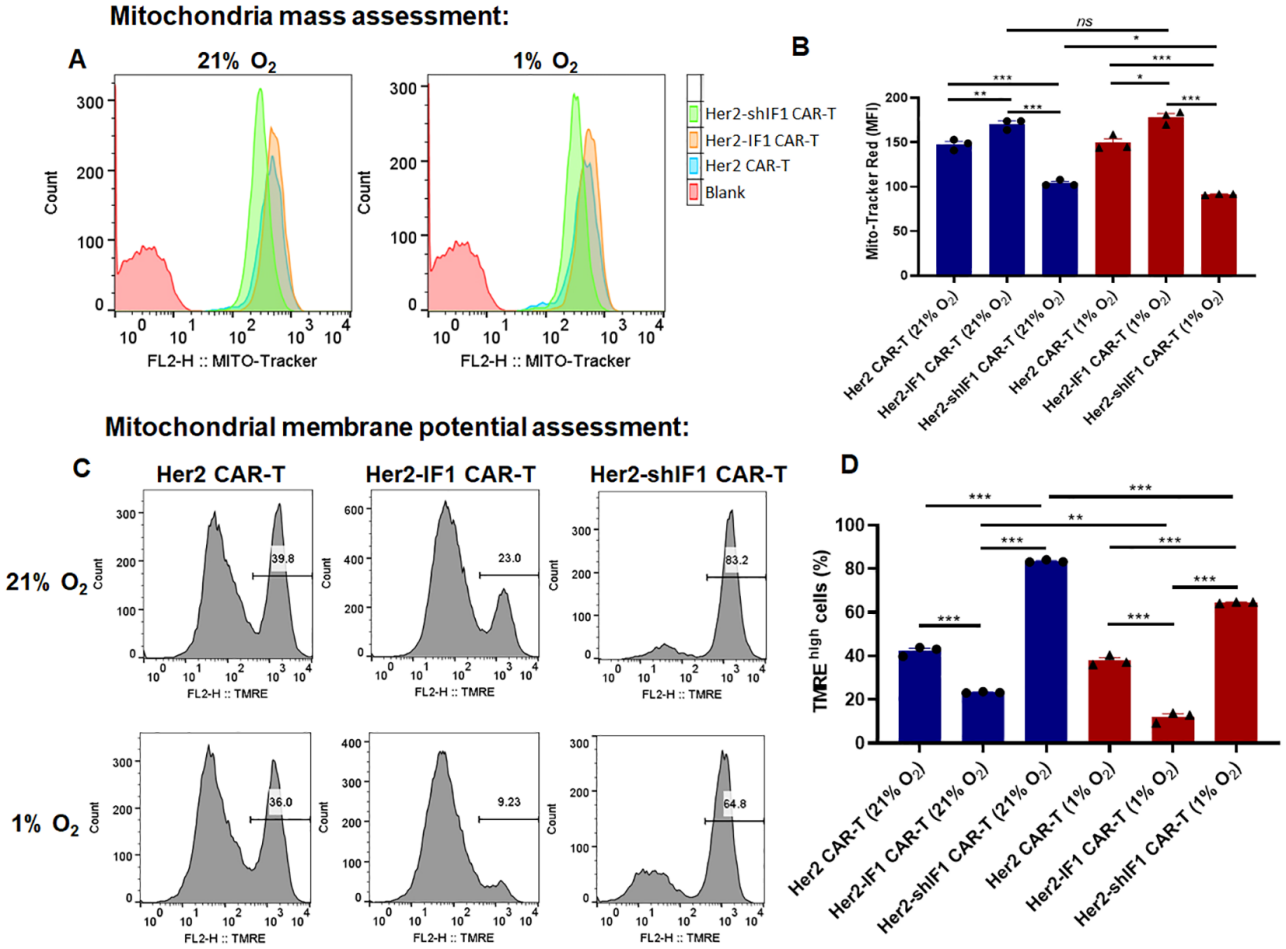
Quantification of mitochondrial mass **(A, B)** and mitochondrial membrane potential (MMP) **(C, D)** in CAR-T cells under normoxic (21% O_2_) or hypoxic (1% O_2_) conditions *in vitro*. The dye Mito-Tracker Red CMXros was used to assess the mitochondria mass, and the dye TMRE was used to assess the change of mitochondria membrane potential. *P < 0.05; **P < 0.01; ***P < 0.001.

**Figure 6:**
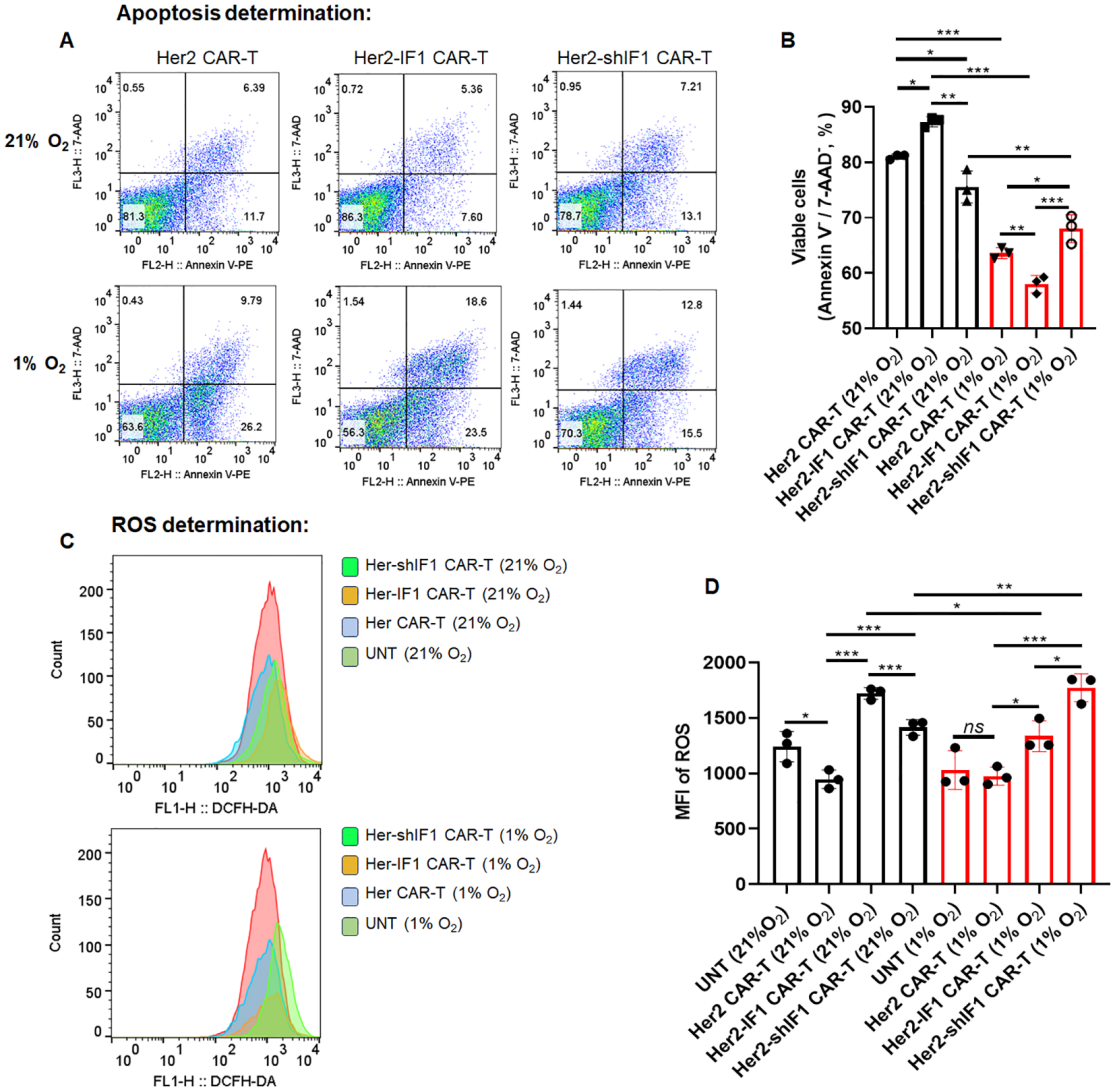
Detection of apoptosis and reactive oxygen species (ROS) levels in CAR-T cells under normoxic (21% O_2_) or hypoxic (1% O_2_) conditions *in vitro*. **(A , B)** the CAR-T cells were stained with Annexin V and 7-AAD to detect the viable cells under different O_2_ concentration for 24 hours. **(C, D)** the CAR-T cells were stained with DCFH-DA for ROS determination after cultivation under different O_2_ concentration for 24 hours. *P < 0.05; **P < 0.01; ***P < 0.001.

To elucidate the contradictory phenotypes of Her2-IF1 CAR-T and Her2-shIF1 CAR-T cells observed *in vitro* versus *in vivo*, we analyzed several key characteristics of these CAR-T cells. First, mitochondrial mass was quantified by flow cytometry. As shown in **Figures 5A, B**, ATPIF1 overexpression increased mitochondrial mass, whereas ATPIF1 knockdown reduced it—indicating a positive correlation between ATPIF1 expression levels and mitochondrial mass. Second, mitochondrial membrane potential (MMP) was assessed. We found that ATPIF1 overexpression decreased MMP, while ATPIF1 knockdown elevated it. Furthermore, MMP was further reduced under hypoxic conditions (1% O_2_; **Figures 5C, D**). This regulatory effect of ATPIF1 on MMP is consistent with previous reports (15–17). To further unravel the dual role of ATPIF1 in modulating CAR-T cell activity *in vitro* and *in vivo*, we determined the apoptosis rate of CAR-T cells stimulated with SKBR3-conditioned medium under different oxygen concentrations. As shown in **Figures 6A, B**, ATPIF1 knockdown increased the apoptotic rate under normoxic conditions (21% O_2_) but enhanced cell survival under hypoxic conditions (1% O_2_), ATPIF1 overexpression exerted the opposite effects. Regarding reactive oxygen species (ROS) production, ATPIF1 overexpression increased ROS levels in CAR-T cells compared to parental Her2 CAR-T cells under 21% O_2_ condition. Notably, ROS levels were universally reduced in Her2 CAR-T and Her2-IF1 CAR-T cells under hypoxic conditions, whereas Her2-shIF1 CAR-T cells exhibited the highest ROS content among the three groups under 1% O_2_ condition (**Figures 6C, D**).

### 3.6 ATPIF1 expression impacts the CAR-T cell migration and survivability in tumor

**Figure 7:**
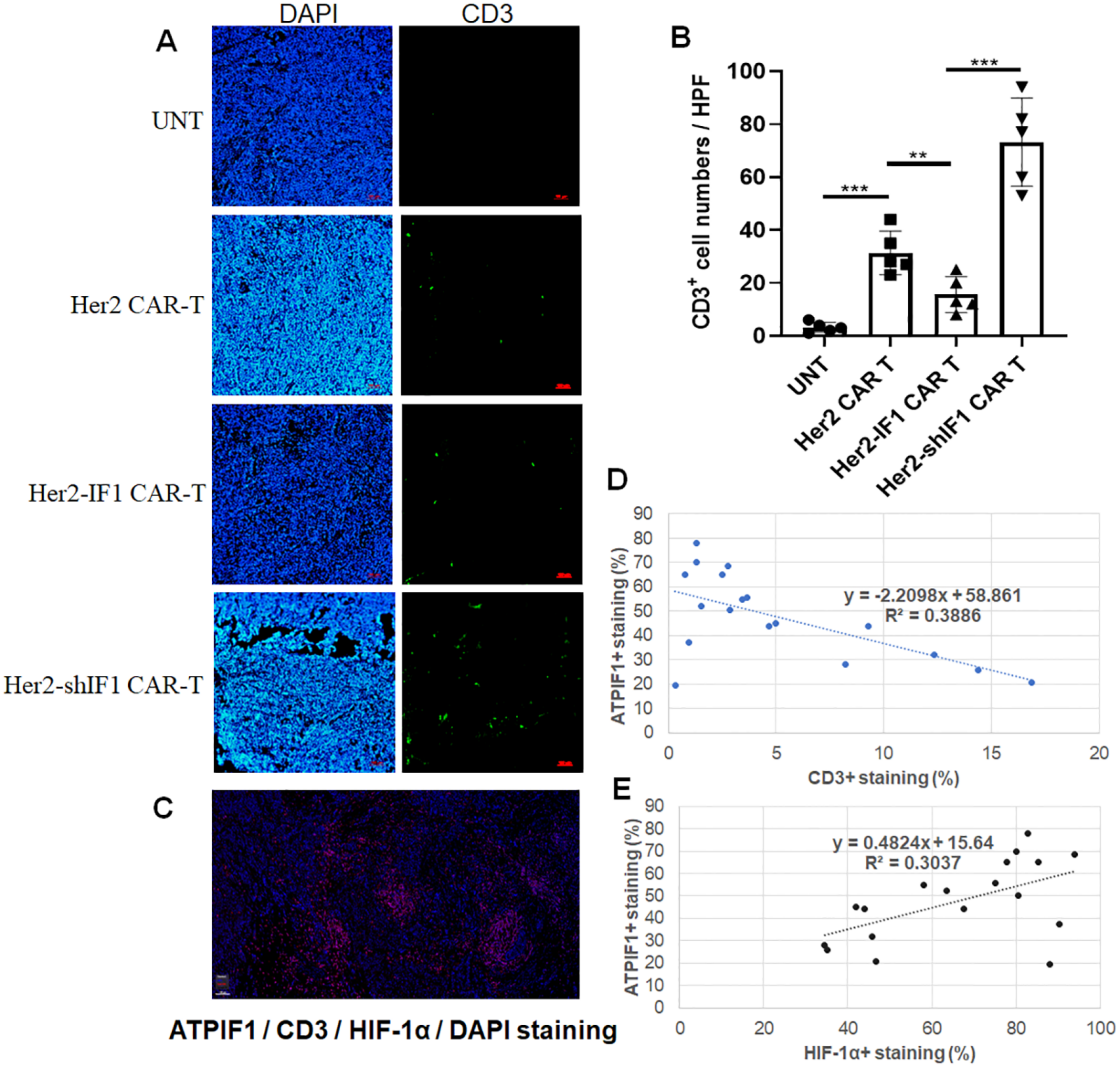
Immunofluorescence and multiplex immunohistochemistry analyses. **(A, B)** Enhanced CD3^+^staining in group of Her2-shIF1 CAR-T in SKBR-3 xenografted tumor tissues. NCG mice were xenografted with SKBR3 cells (n=5 in each group); when tumors reached ~100 mm³, mice were intravenously injected with untransfected T cells (UNT), Her2 CAR-T, Her2-IF1 CAR-T, or Her2-shIF1 CAR-T cells. Four days later, Mice were euthanized, and tumors were excised for immunofluorescence staining. **(C)** Multiplex immunohistochemistry staining of 18 formalin-fixed paraffin-embedded (FFPE) human breast cancer tissue sections, showing ATPIF1 (yellow), CD3 (green), HIF-1α (orange), and DAPI (blue). **(D, E)** Quantitative analysis of the correlation between CD3^+^staining and ATPIF1^+^cells **(D)**, and between HIF-1α^+^and ATPIF1^+^cells **(E)** from the results of Multiplex immunohistochemistry staining in **(C)** respectively. **P<0.01, ***P<0.001.

The aforementioned results indicated that ATPIF1 modulates the survival capacity of CAR-T cells under distinct oxygen conditions. Thus, the enhanced *in vivo* antitumor activity of Her2-shIF1 CAR-T cells might be attributed to their improved survival rate within solid tumors, which typically exhibit a hypoxic microenvironment. To test this hypothesis, we administered CAR-T cells to tumor-xenografted mice and employed immunofluorescence staining to evaluate CAR-T cell infiltration and survival within tumors. As illustrated in **Figures 7A, B**, Her2-shIF1 CAR-T cells showed the highest accumulation in solid tumors, as evidenced by the greatest number of CD3^+^-stained cells per high-power field (HPF). Concurrently, *in vitro* transwell assays were performed (**Supplementary Figure 2**), revealing that Her2-shIF1 CAR-T cells exhibited the strongest migratory capacity under hypoxic conditions (1% O_2_). In contrast, Her2-IF1 CAR-T cells displayed optimal mobility under normoxic conditions (21% O_2_), indicating that ATPIF1 exerts opposing effects on CAR-T cell migration depending on the oxygen concentration. To further explore the correlation between ATPIF1 expression and T cell migration in clinical tumors, multi-color immunofluorescence staining was conducted on human breast cancer tissues (**Figure 7C**). We found that ATPIF1 expression was negatively correlated with CD3^+^T cell infiltration, while HIF-1α expression was positively correlated with ATPIF1 levels (**Figures 7D, E**). These results suggest that higher ATPIF1 expression in breast cancer tissues is associated with reduced CD3^+^T cell infiltration and survival.

### 3.7 ATPIF1 knockdown increased the mtDNA leakage and activated STING signal pathway

**Figure 8:**
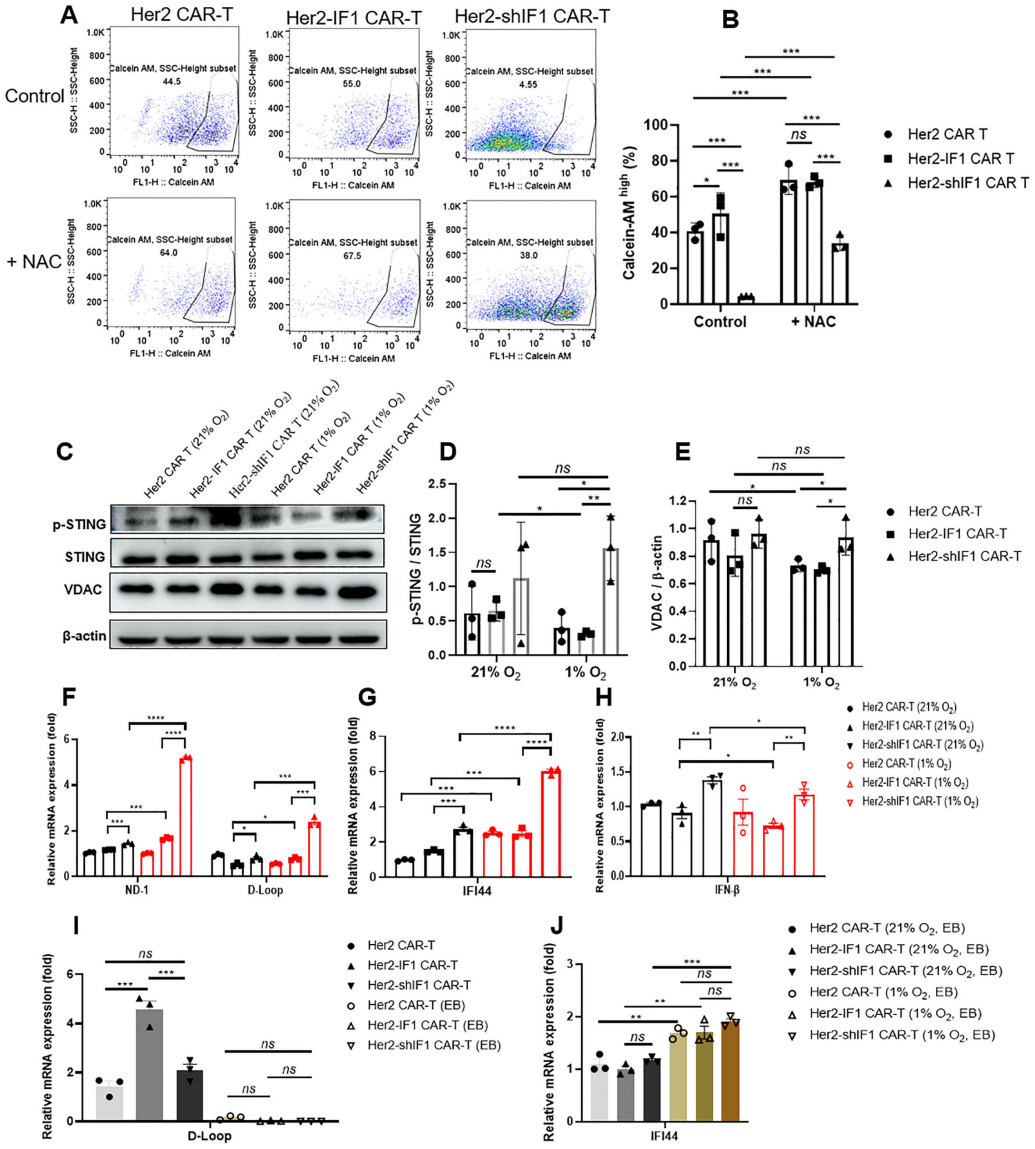
ATPIF1 knockdown induces mPTP opening, mtDNA leakage, and STING signaling pathway activation. **(A, B)** Flow cytometric detection of mPTP opening using Calcein-AM staining. The Her2 CAR-T, Her2-IF1 CAR-T and Her-shIF1 CAR-T cells were treated with or without N-Acetylcysteine (NAC, 10 mM) for 24 hours, and then stained with Calcein-AM, flow cytometry was used to detect the content of Calcein-AM, which indicated the levels of mPTP opening. **(C)** Western blot analysis of p-STING, total STING, and VDAC protein expression. **(D)** Quantification of p-STING relative to total STING. **(E)** Quantification of VDAC relative to β-actin (loading control). **(F–H)** RT-qPCR analysis of cytoplasmic mtDNA (ND-1 and D-loop) and mRNA levels of IFI44 and IFN-β. Cytoplasmic RNA was extracted for detection. **(I)** Generation of ρ^0^CAR-T cells via ethidium bromide (EB) treatment for 2 weeks. Total mtDNA was extracted to detect D-loop levels. **(J)** RT-qPCR analysis of IFI44 mRNA expression. *P < 0.05; **P < 0.01; ***P < 0.001; ****P<0.0001.

Mitochondrial permeability transition pore (mPTP) opening is suspected to promote cellular apoptosis, and Calcein-AM is a commonly used dye for detecting mPTP opening—with reduced intracellular Calcein-AM fluorescence indicating increased mPTP opening. As shown in **Figures 8A, B**, Her2-shIF1 CAR-T cells exhibited a significant decrease in Calcein-AM fluorescence intensity, suggesting enhanced mPTP opening, and this were might related with the ROS, as the elimination of ROS with N-Acetylcysteine (NAC) treatment could increase the Calcein-AM ratio. Western blot analysis revealed that Her2-shIF1 CAR-T cells had upregulated protein expression of phosphorylated STING (p-STING), total STING, and VDAC (**Figures 8C–E**), indicating activation of the STING signaling pathway. Previous studies have reported that mPTP opening can induce mitochondrial DNA (mtDNA) leakage, which further activates the STING pathway and subsequently enhances *in vivo* T cell infiltration (18, 19). To explore this mechanism, we extracted cytoplasmic mtDNA and detected the levels of ND-1 and D-loop (mtDNA-specific markers) using qPCR; we also quantified the mRNA expression of IFI44 and IFN-β (STING pathway downstream targets) via qPCR. As depicted in **Figures 8F–H**, the levels of ND-1, D-loop, IFI44, and IFN-β were all significantly upregulated in Her2-shIF1 CAR-T cells, suggesting that the increased STING phosphorylation in these cells may be associated with mtDNA leakage. To further validate this causal link, we depleted mtDNA from CAR-T cells using ethidium bromide (EB) to generate ρ^0^cells (**Figure 8I**). Following mtDNA depletion, there were no significant differences in the protein expression of p-STING and VDAC, or in the mRNA expression of IFI44, among Her2 CAR-T, Her2-IF1 CAR-T, and Her2-shIF1 CAR-T cells (**Figure 8J**; **Supplementary Figure 3**).

### 3.8 Inhibition of STING decreased the migration of CAR-T cells and antitumor activity *in vivo*

**Figure 9:**
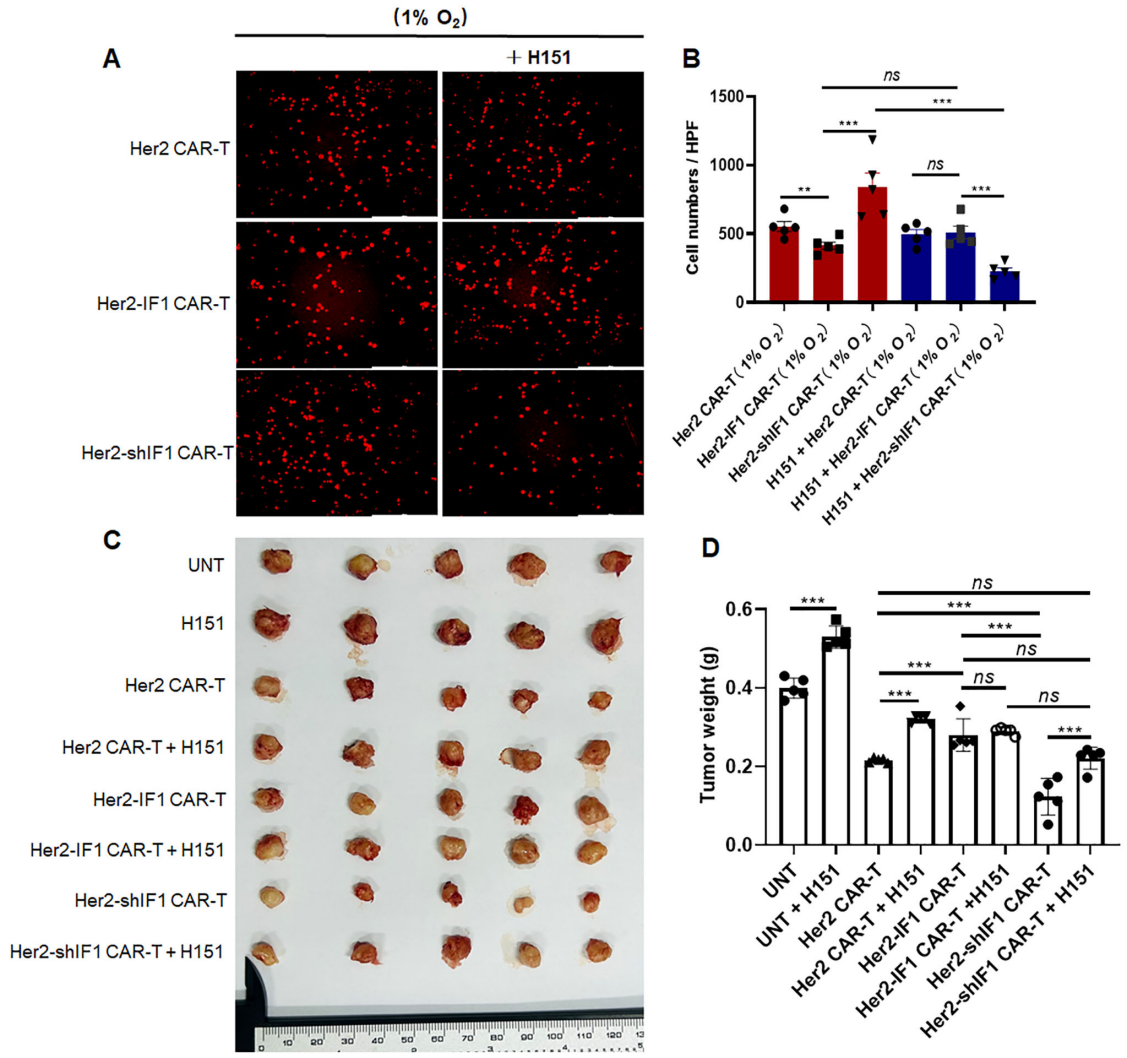
The enhanced *in vivo* antitumor activity of Her2-shIF1 CAR-T cells is dependent on STING activation. **(A, B)** Transwell migration assay of CAR-T cells under hypoxic conditions (1% O_2_) with or without H151 inhibition. CAR-T cells were stained with Cell-Tracker Red CMTPX and seeded into the upper Transwell chamber. Migrated cells in the lower chamber were visualized via fluorescence microscopy, with five random fields per well imaged for quantification. **(C, D)** Excised tumor size and weight following treatment with different CAR-T cells, either alone or in combination with H151. NCG mice were xenografted with SKBR3 cells (n=5 in each group). When tumors reached ~100 mm³, mice were intravenously injected with untransfected T cells (UNT), Her2 CAR-T, Her2-IF1 CAR-T, or Her2-shIF1 CAR-T cells (1x10^6^ CAR-T cells per mouse); or treated with or without H151 via intraperitoneal injection (7 mg/kg) as indicated. Two weeks later, the mice were euthanized, tumors were excised and weighed. **P < 0.01; ***P < 0.001.

H151, a specific inhibitor of STING was used to investigate its impact on CAR-T cell migration. Consistent with our prior findings (**Supplementary Figure 2**), Her2-shIF1 CAR-T cells exhibited the strongest migratory capacity under hypoxic conditions (1% O_2_), and this enhanced migration was significantly abrogated following H151 treatment (**Figures 9A, B**). These results indicate that activation of the STING signaling pathway is responsible for the improved *in vitro* migration of Her2-shIF1 CAR-T cells. To further validate this observation, we conducted *in vivo* animal experiments. As depicted in **Figures 9C, D**, NCG mice were xenografted with SKBR3 breast cancer cells and then administered CAR-T cells either alone or in combination with H151. Consistent with the results shown in **Figure 4**, Her2-IF1 CAR-T cells displayed reduced tumor-inhibitory efficacy compared to parental Her2 CAR-T cells, while Her2-shIF1 CAR-T cells exerted the most potent antitumor activity among the three groups. Notably, H151 treatment attenuated the antitumor efficacy of CAR-T cells, underscoring the critical role of the STING signaling pathway in regulating CAR-T cell antitumor function.

## 4 Discussion

In this study, we modulated the expression of ATPIF1 in Her2-targeted CAR-T cells and systematically investigated its impact on the antitumor efficacy of these cells. As anticipated, ATPIF1 overexpression significantly enhanced the *in vitro* antitumor activity of Her2-targeted CAR-T cells against breast cancer, characterized by improved tumor lysis capacity, increased secretion of IL-2 and IFN-γ, and even prolonged *in vivo* persistence of CAR-T cells. Surprisingly, however, the *in vivo* antitumor effects were reversed, Her2-IF1 CAR-T cells exhibited the weakest tumor-inhibitory efficacy compared to parental Her2 CAR-T and Her2-shIF1 CAR-T cells. In contrast, Her2-shIF1 CAR-T cells displayed a phenotype opposite to that of Her2-IF1 CAR-T cells, highlighting that ATPIF1 expression exerts bidirectional regulatory effects on the antitumor activity of CAR-T cells.

It is well established that ATPIF1 plays a critical role in regulating mitochondrial cristae density (15, 20). Consistent with this, our data showed that ATPIF1 overexpression increased mitochondrial mass in Her2-IF1 CAR-T cells (**Figures 5A, B**). Consequently, under oxygen-replete conditions, Her2-IF1 CAR-T cells exhibited enhanced antitumor activity and elevated oxidative phosphorylation (OXPHOS) levels, accompanied by prolonged *in vivo* persistence. Previous studies have reported that enhanced oxygen consumption rate (OCR) capacity can improve CAR-T cell persistence (21), which is consistent with our earlier findings in CD19-targeted CAR-T cells (10). In contrast, ATPIF1 knockdown impaired the *in vitro* antitumor activity of Her2-targeted CAR-T cells and reduced their OXPHOS capacity. Her2-shIF1 CAR-T showed the slowest proliferation and lowest tumor lytic activities as compared to that of Her2 CAR-T and Her2-IF1 CAR-T, this was the indication of functional defect of T cells. However, the antitumor performance of Her2-IF1 CAR-T and Her2-shIF1 CAR-T cells was completely reversed in the *in vivo* setting.

To unravel the underlying mechanism, the bidirectional function of ATP synthase—capable of both synthesizing and hydrolyzing ATP—may serve as a key regulatory node, as this dual activity is dependent on mitochondrial membrane potential (MMP) (8, 9). Gu et al. reported that ATPIF1 is critical for mitochondrial cristae biogenesis (22) and the functional regulation of ATP synthase (20). Specifically, ATPIF1 overexpression not only increases the number of mitochondrial cristae but also inhibits ATP synthase activity, which may contribute to reduced ATP production (23) (**Supplementary Figure 4**) and diminished MMP (17) (**Figures 5C, D**). Theoretically, ATPIF1 knockdown should relieve this inhibitory constraint on ATP synthase, thereby promoting increased ATP generation. However, under normoxic conditions (21% O_2_), the ATP level in Her2-shIF1 CAR-T cells was not significantly higher than that in Her2-IF1 CAR-T cells. This suggests the involvement of additional regulatory factors, for example, ATPIF1 depletion may reduce mitochondrial cristae mass (a structural determinant of ATP production efficiency), offsetting the potential gain from enhanced ATP synthase activity. Notably, consistent with previous reports (17), the MMP in Her2-shIF1 CAR-T cells was higher than that in parental Her2 CAR-T cells. Intriguingly, under hypoxic conditions (1% O_2_) *in vitro*, the ATP content in Her2-shIF1 CAR-T cells was significantly elevated, a phenomenon that warrants further investigation to clarify its molecular basis.

Then why did Her2-IF1 CAR-T and Her2-shIF1 CAR-T cells exhibit opposing phenotypes *in vivo*? Under normoxic conditions (21% O_2_), ATPIF1 enhanced oxygen consumption rate (OCR), reduced apoptosis, and promoted the acquisition of a memory phenotype in CAR-T cells (**Figures 3D, F**). However, under hypoxic conditions, hypoxia-inducible factor 1α (HIF-1α) can further upregulate ATPIF1 expression (23). Consequently, the excessive elevation of ATPIF1 may further inhibit ATP synthase activity, leading to a more pronounced reduction in mitochondrial membrane potential (MMP), as well as increased cellular apoptosis (**Figures 6A, B**) and exhaustion (**Figure 3G**). In contrast, ATPIF1 knockdown under hypoxia relieves the inhibitory constraint on ATP synthase, which can then reverse proton flux, which pumping protons from the mitochondrial matrix to the intermembrane space to maintain the MMP. This, in turn, enhances the survival of Her2-shIF1 CAR-T cells. Consistent with this mechanism, Her2-shIF1 CAR-T cells exhibited the highest MMP among the tested groups, and previous studies have reported that higher MMP correlates with superior *in vivo* antitumor efficacy (5).

In the tumor microenvironment (TME) of solid tumors, T cells frequently undergo mitochondrial dysfunction and impaired electron transport chain (ETC) activity (24, 25). A genome-wide insertional mutagenesis screen conducted by Chen W et al. demonstrated that under conditions of ETC impairment, knockdown of ATPIF1 expression promotes T cell survival (26). Thus, in the context of solid tumors, appropriate downregulation of ATPIF1 may help relieve the inhibitory constraint on ATP synthase, enabling it to maintain mitochondrial membrane potential (MMP) stability via ATP hydrolysis, an effect that favors T cell survival and preserves antitumor activity. However, complete ablation of ATPIF1 could lead to unregulated ATP synthase hydrolytic activity, resulting in excessive intracellular ATP depletion, abnormal membrane hyperpolarization, and mitochondrial hyperpolarization (27). This, in turn, ultimately impairs T cell function and diminishes antitumor efficacy (10, 11).

The enhanced infiltration of Her2-shIF1 CAR-T cells may be attributed to increased intracellular ATP levels and STING phosphorylation. Ledderose C et al. reported a correlation between ATP content and T cell infiltration into tumors (28), and given that Her2-shIF1 CAR-T cells exhibit higher ATP levels under hypoxic conditions (**Supplementary Figure 4**), this likely contributes to their improved infiltration capacity. Additionally, activation of the STING pathway is another key driver of enhanced infiltration in Her2-shIF1 CAR-T cells. Accumulating evidence indicates that higher STING expression correlates with more efficient T cell infiltration into solid tumors (18, 19), suggesting that mtDNA leakage-induced STING activation may promote the infiltration of Her2-shIF1 CAR-T cells. To further strengthen the connection of STING activation and CAR-T cells infiltration, we stained the CD3^+^ and p-STING, it seems that the CD3^+^ T cells had well colocalization with p-STING as shown in **Supplementary Figure 5**. However, the mechanism underlying increased mtDNA leakage following ATPIF1 knockdown remains unclear.

mtDNA is known to be released through various channels, including VDAC oligomeric pores (29), BAK/BAX pores (30), and other alternative pathways. Esparza-Moltó PB et al. demonstrated that ATPIF1 overexpression upregulates VDAC expression (31), implying that ATPIF1 knockdown is unlikely to facilitate mtDNA release via VDAC oligomeric pores. While BAK/BAX pores are associated with apoptotic cell death (30), ATPIF1 knockdown enhances CAR-T cell survival under hypoxia, making BAK/BAX pores also an implausible route for the increased mtDNA leakage in Her2-shIF1 CAR-T cells. Recently, emerging evidence has identified the ATP synthase c subunit as a novel pore mediating mtDNA release (32, 33). If the ATP synthase supercomplex indeed functions as an mtDNA release channel, ATPIF1 depletion may augment mtDNA leakage through this pathway, an intriguing possibility that requires further experimental validation, this is the limitation of this study, as we cannot fully to elucidate the underlying mechanism to explain the link between ATPIF1 knockdown and mtDNA leakage. Also, as Her2-shIF1 CAR-T cells showed increased mPTP opening, whereas the percentage of viable cells under 1% O_2_ condition was higher than that of Her2-IF1 CAR-T cells, thus it is seems contradictory for mPTP opening and increased cell apoptosis. Therefore, is it possible for that transient or low-level mPTP opening for mtDNA release is beneficial for cell survival, whereas a full-blown mPTP opening might induce cell apoptosis and was detrimental? This needs further investigation. Also, it was reported that increased mtDNA release and STING activation might increase the risk of systemic inflammation or toxicity (34), such as systemic lupus erythematosus (SLE), therefore the Her2-shIF1 CAR-T might increase the cytokine-related adverse effects, a strategy for hypoxic induction of ATPIF1 knockdown expression should be developed to avoid this potential side effect in CAR-T design.

Notably, our study found no correlation between the enhanced *in vivo* persistence of Her2-IF1 CAR-T cells and improved antitumor efficacy compared to parental Her2 CAR-T cells, indicating that CAR-T cell persistence is not the decisive factor for antitumor outcomes. Minn I et al. utilized Positron Emission Tomography (PET) to track CAR-T cells in mice and observed no absolute correlation between the number of peripheral blood CAR-T cells and intratumoral CAR-T cell accumulation in some cases (35). In other words, a higher number of peripheral CAR-T cells did not necessarily translate to greater intratumoral accumulation, highlighting that the infiltration and survival of CAR-T cells within the solid tumor microenvironment are more critical for achieving optimal antitumor efficacy.

In summary, this study demonstrates that ATPIF1 expression exerts a profound influence on the antitumor function of CAR-T cells. The bidirectional regulatory role of ATPIF1 in modulating CAR-T antitumor activity is particularly noteworthy, as it provides valuable insights for the development of novel strategies to enhance the therapeutic efficacy of CAR-T cells. Given the complexity of the tumor microenvironment (TME), characterized by hypoxia and hypoglycemia, robust *in vitro* proliferation or antitumor activity of CAR-T cells does not necessarily translate to superior *in vivo* performance. This partly explains why CAR-T therapy has achieved remarkable success in hematological malignancies but remains suboptimal in solid tumors. Importantly, conventional *in vitro* assays for evaluating CAR-T activity may not always be reliable for predicting *in vivo* antitumor outcomes. Thus, for the further advancement of CAR-T therapy, careful design and interpretation of *in vitro* experiments are imperative, with full consideration of the physiological relevance to the *in vivo* TME.

## Data Availability

The original contributions presented in the study are included in the article/**Supplementary Material**. Further inquiries can be directed to the corresponding authors.
