# Supplementary material for: The critical role of Atpif1 in Her2-targeted CAR-T cell therapy for solid tumor via modulation of metabolism and mtDNA-STING signal pathway
Source: Front Immunol. 2026 Mar 2;17:1733753. doi: 10.3389/fimmu.2026.1733753 (PMC12989527; doi:10.3389/fimmu.2026.1733753)
Supplement: Supplementary file 1 [file DataSheet1.pdf]

Figure supplement

Figure S1 ATPIF1 was overexpressed successfully in CAR-T cells as determined western blot. (***Notes***: The overexpression of ATPIF1 in cells could generated a larger band in WB graph, which was in consist with the published paper in Journal of Biological Chemistry (J Biol Chem, 2022; 298(5):101858. doi: 10.1016/j.jbc.2022.101858). The total grey value of ATPIF1 in each WB line was calculated and compared to that of β-actin, and obtain the ratio in B)

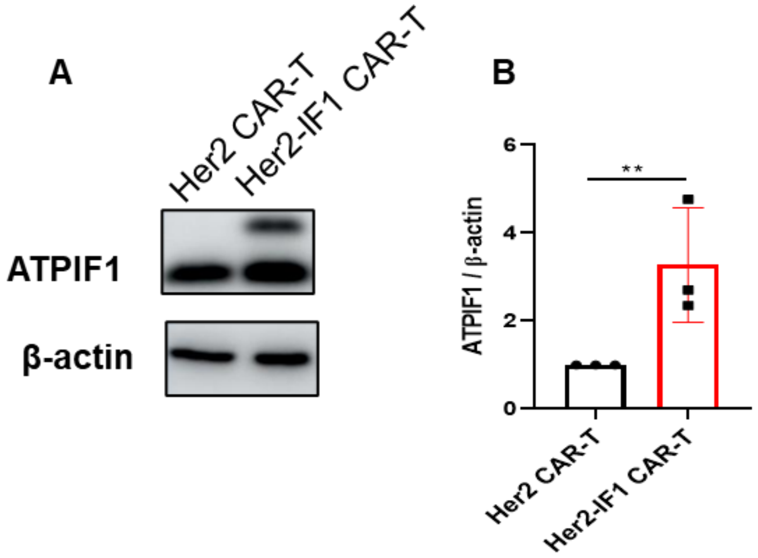

Figure S2 ATPIF1 expression had great impact on CAR-T cells migration in vitro under different oxygen concentration.

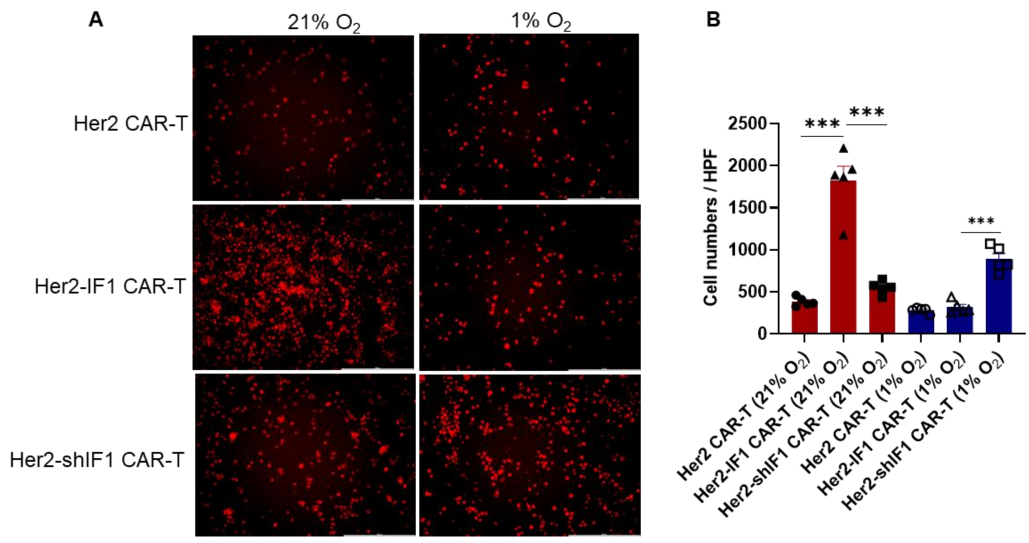

Figure S3 The p-STING and VDAC expression had no significant difference after the depletion of mtDNA with EB treatment as determined by western blot.

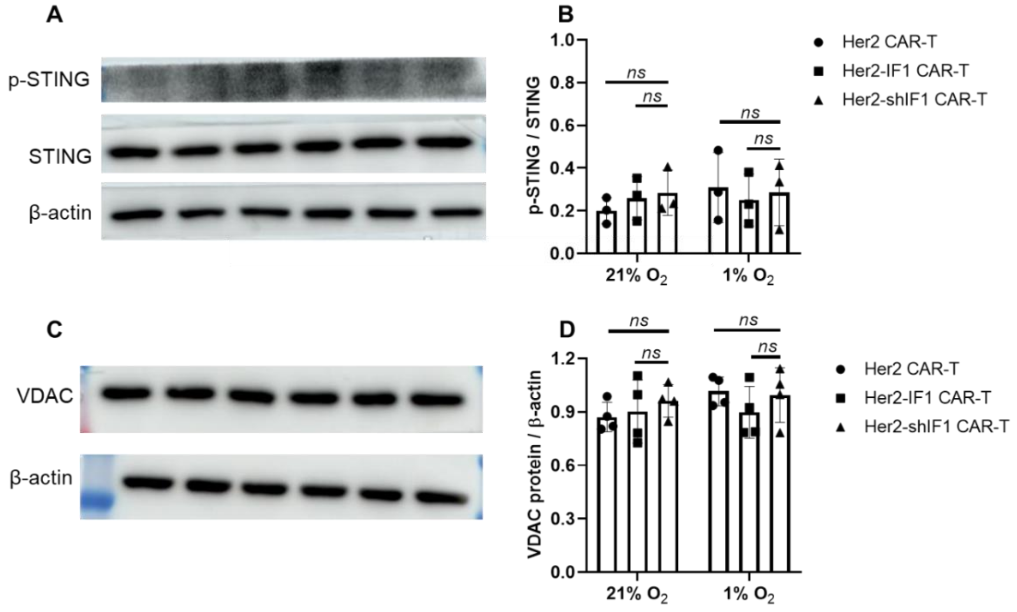

Figure S4 ATP content determination in CAR-T cells under different oxygen concentration *in vitro*.

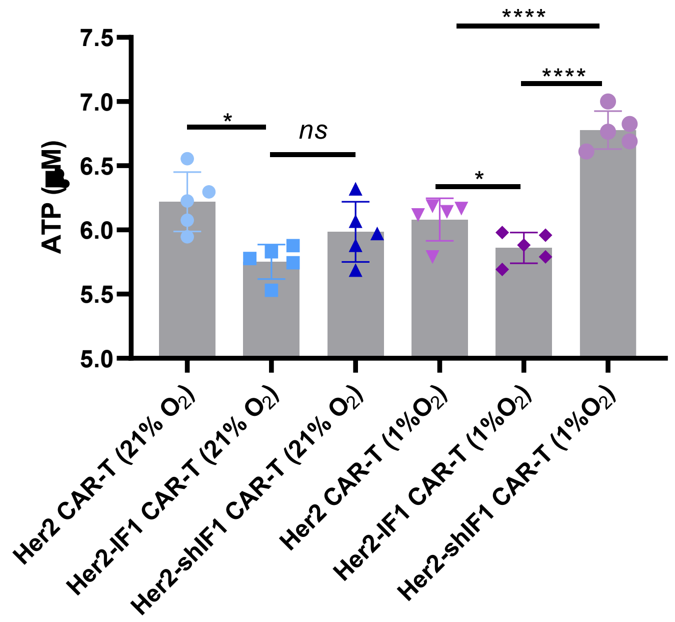

Figure S5 The staining of CD3+ and p-STING with the tumor tissues in Figure 7A.

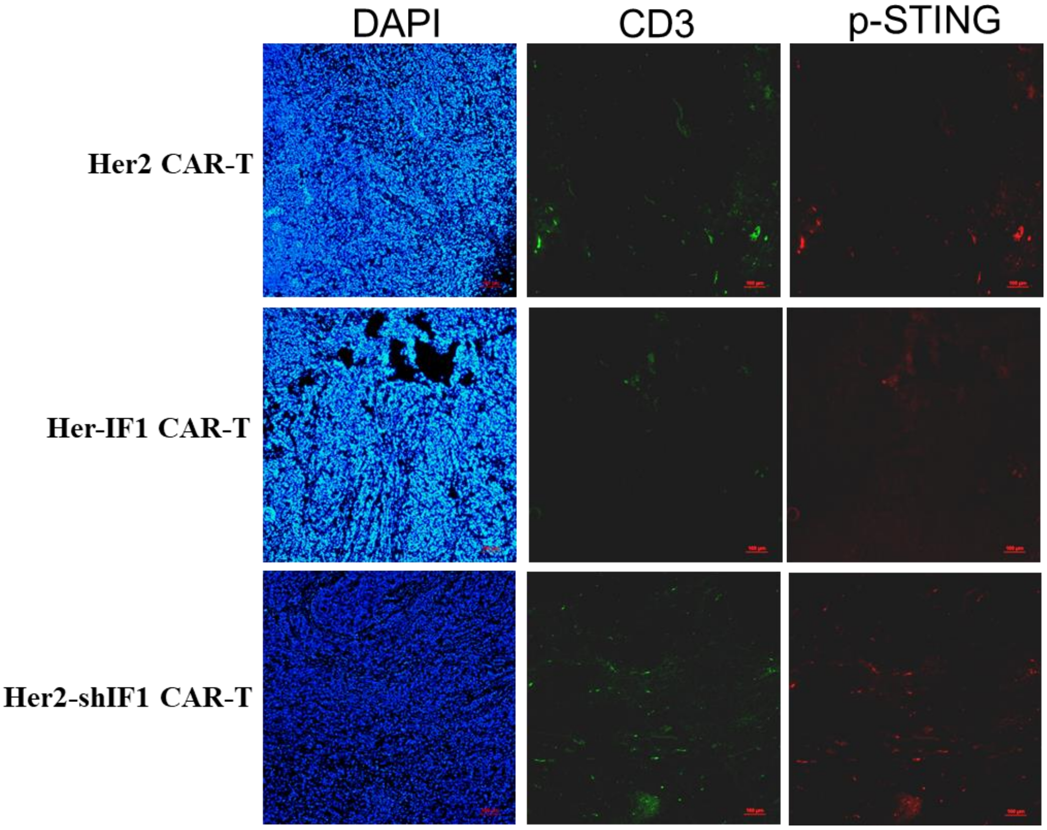

Table S1 Primers used in the manuscript

**Names**	**Primer sequences (5'→3')**
ATPIF1	F: ACGATATTTCCGAGCACAGAG
	R: TCTGCAGACGCTCAATCTCC
β-actin	F: CATGTACGTTGCTATCCAGGC
	R: CTCCTTAATGTCACGCACGAT
ND-1	F: CCCTAAAACCCGCCACATCT
	R: GAGCGATGGTGAGAGCTAAGGT
D-Loop	F: CTATCACCCTATTAACCACTCA-
	R: TTCGCCTGTAATATTGAACGTA
IFI44	F: GATGTGAGCCTGTGAGGTCC
	R: CTTTACAGGGTCCAGCTCCC
IFN-β	F: ACGCCGCATTGACCATCTAT
	R: GTCTCATTCCAGCCAGTGCT
GUSB	F: GAAAATACGTGGTTGGAGAGCTCATT
	R: CCGAGTGAAGATCCCCTTTTTA

The following graphs were the Western blot results of replicates:

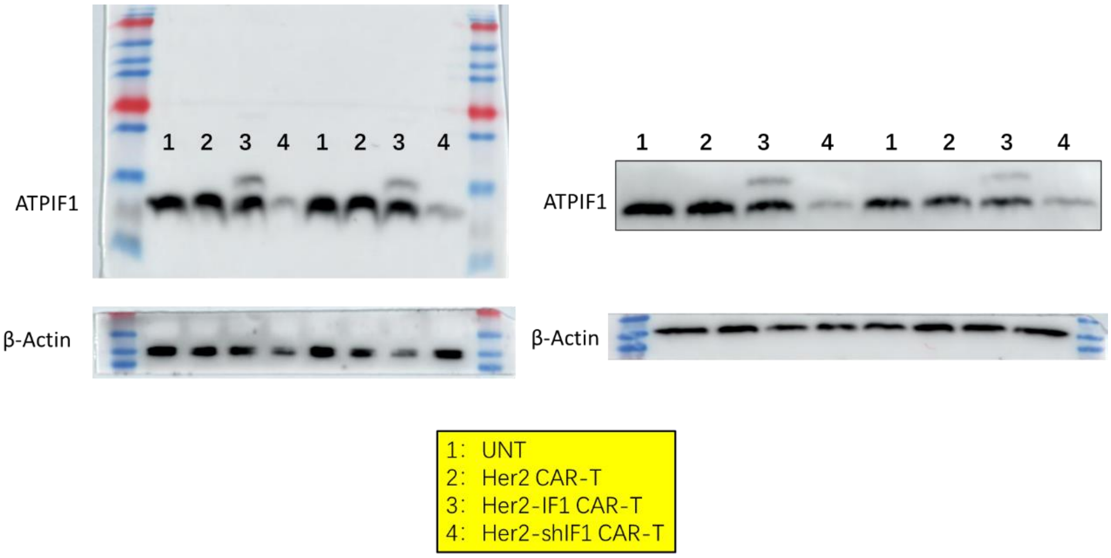

The Western Blot of ATPIF1 in CAR-T cells.

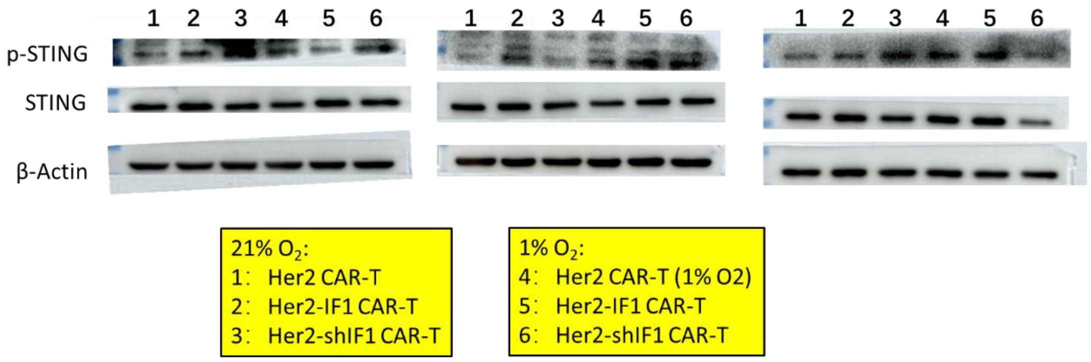

The Western Blot of STING, p-STING in CAR-T cells under 21% O_2 and 1% O_2 concentration.

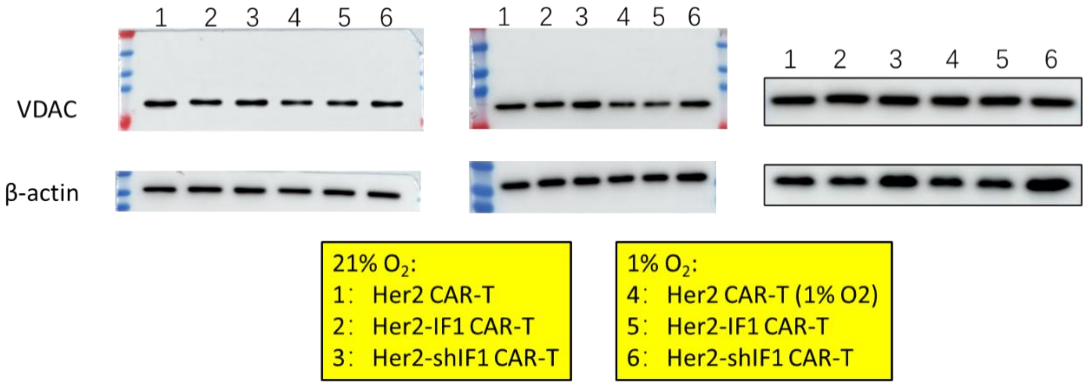

The Western Blot of VDAC in CAR-T cells under 21% O2 and 1% O2 concentration.

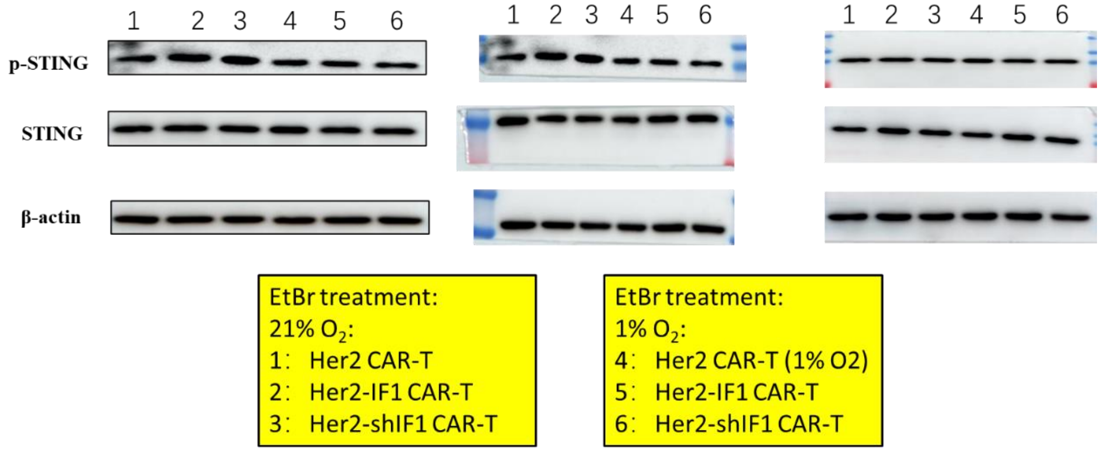

The Western Blot of STING, p-STING in CAR-T cells under 21% O2 and 1% O2 concentration after EtBr treatment.

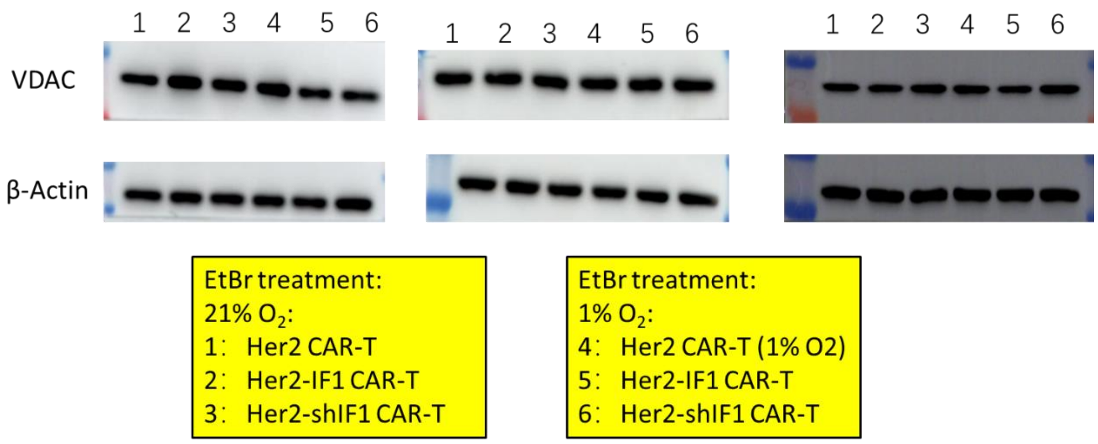

The Western Blot of VDAC in CAR-T cells under 21% O2 and 1% O2 concentration after EtBr treatment.

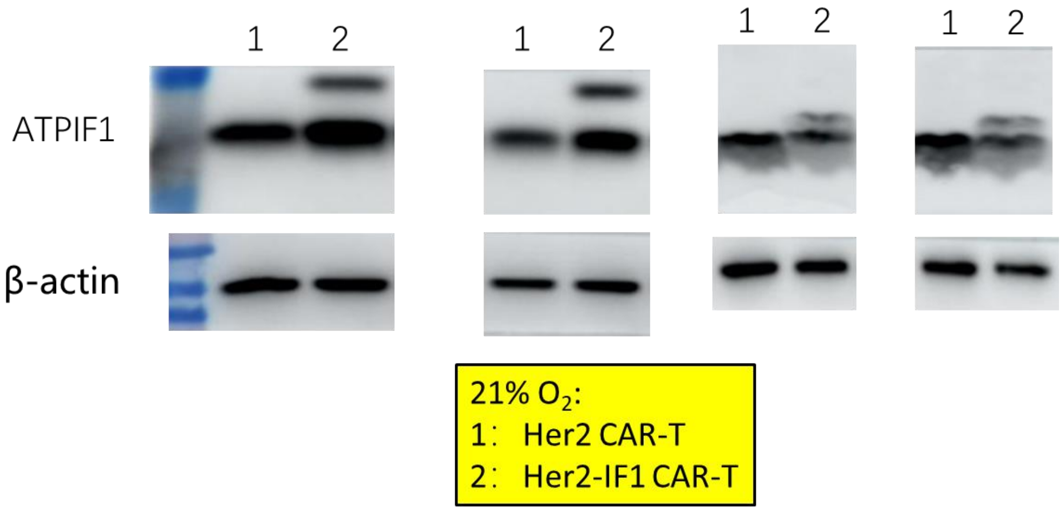

The Western Blot of ATPIF1 in CAR-T cells. The location of ATPIF1 expression was similar with the reported of Guo L in J Biol Chem, 2022; 298(5):101858. doi: 10.1016/j.jbc.2022.101858.
